# Regulation of Circular RNA CircNFATC3 in Cancer Cells Alters Proliferation, Migration, and Oxidative Phosphorylation

**DOI:** 10.3389/fcell.2021.595156

**Published:** 2021-03-19

**Authors:** Thasni Karedath, Fatima M. Al-Dasim, Ikhlak Ahmed, Albandary Al-Qurashi, Afsheen Raza, Simeon Scott Andrews, Ayeda Abdulsalam Ahmed, Yasmin Ali Mohamoud, Said Dermime, Joel A. Malek

**Affiliations:** ^1^Weill Cornell Medicine-Qatar, Doha, Qatar; ^2^Sidra Medicine, Doha, Qatar; ^3^National Center for Cancer Care and Research, Hamad Medical Corporation, Doha, Qatar

**Keywords:** circRNA, RNA-seq, siRNA, circRNA mini vector, migration, invasion, oxidative phosphorylation

## Abstract

Circular RNAs were once considered artifacts of transcriptome sequencing but have recently been identified as functionally relevant in different types of cancer. Although there is still no clear main function of circRNAs, several studies have revealed that circRNAs are expressed in various eukaryotic organisms in a regulated manner often independent of their parental linear isoforms demonstrating conservation across species. circNFATC3, an abundant and uncharacterized circular RNA of exon 2 and 3 from *NFATC3*, was identified in transcriptomic data of solid tumors. Here we show that circNFATC3 gain- and loss-of-function experiments using RNAi-mediated circRNA silencing and circular mini vector-mediated overexpression of circularized constructs in breast and ovarian cancer cell lines affect molecular phenotypes. The knockdown of circNFATC3 induces a reduction in cell proliferation, invasion, migration, and oxidative phosphorylation. Gain-of-function of circNFATC3 in MDA-MB-231 and SK-OV-3 cells show a significant increase in cell proliferation, migration, and respiration. The above results suggest that circNFATC3 is a functionally relevant circular RNA in breast and ovarian cancer.

## Introduction

Circular RNAs (circRNAs) were previously considered transcriptional byproducts, however, they drew attention after the functional characterization of a few circular RNAs such as *CDR1-AS* (*ciRS-7*), *SRY*, and *HIPK3* (Wilusz and Sharp, 2013; Jeck et al., 2013; Salzman, 2016). *ciRS-7*, one of the initially identified circRNAs and is known for its ability to sponge microRNAs, conceptually changed the understanding of the mechanisms of circRNA and RNA-mediated gene regulation (Hansen et al., 2013). The wide use of high throughput sequencing analysis has been instrumental in identifying novel circular RNAs in different disease phenotypes and tissues (Wang et al., 2014; Szabo and Salzman, 2016). circRNAs are formed during pre-mRNA splicing by non-canonical order which is referred to as backsplicing or head-to-tail splicing where a branch point upstream of an exon attacks a downstream splice donor. This could happen with a single exon or multiple exons resulting in multi-exonic circRNAs. The backsplicing can be induced by the presence of intronic inverted repeat sequences such as *Alu* repeats in flanking introns, which hybridize and bring the ends of relevant exons in proximity forming the circular splice junction (Sibley et al., 2016).

Circular RNAs have attracted increasing attention from cancer researchers as potential biomarkers due to their highly stable nature (Ahmed et al., 2016; Zheng et al., 2016; Karedath et al., 2019a). circRNAs are differentially regulated in human cancers including breast, prostate, brain, bladder, colorectal, ovarian, liver, and kidney as well as hematological malignancies thus it is evident that circRNAs have a significant role in cancer pathogenesis and likely to affect several hallmarks of cancer (Kristensen et al., 2018; Su et al., 2019). Understanding the functional role of circular RNA in cancer cell invasion, migration, and tumor suppression provide new insights into oncogenesis, cancer detection, and prevention which are still elusive. We previously identified circANKRD12 which is a highly expressed circRNA that regulates tumor invasion and migration in breast and ovarian cancer cells (Karedath et al., 2019a). circNFATC3 was identified by our group as a highly expressed circRNA in metastatic ovarian cancer (Ahmed et al., 2016). The parental gene of circNFATC3 belongs to *NFAT* gene family which was first identified in immune cells (Jain et al., 1993; McCaffrey et al., 1993; Urso et al., 2019) and has been associated with malignancies and tumor progression (Jauliac et al., 2002; Mancini and Toker, 2009).

*NFATC3* (nuclear factor of activated T cells 3) is a DNA-binding transcription complex consisting of a preexisting cytosolic component that translocate to the nucleus upon T cell receptor (TCR) stimulation. *NFATC3* plays an important role in retaining stemness *via NFATC3/OCT4* signaling and its overexpression increases tumorigenesis in oral cancer (Lee et al., 2016). Unlike its parental gene, circNFATC3 remains functionally uncharacterized in cancer. Owing to its abundance in tumor tissues, circNFATC3 might be involved in regulating tumor progression and invasion. Our research focuses mainly on the functional characterization of circNFATC3 in cancer cells by conducting circNFATC3 loss- and gain-of-function studies.

## Materials and Methods

### Cell Lines and Treatment

Breast cancer cell lines—MDA-MB-231 (ATCC^®^ HTB-26^TM^), MCF7 (ATCC^®^ HTB-22^TM^)—and breast normal cell line—MCF 10A (ATCC^®^ CRL-10317^TM^)-, Primary Mammary Epithelial Cells; Normal, Human (HMEC) (ATCC^®^ PCS-600-010^TM^), ovarian cancer cell lines—PA-1 (ATCC^®^ CRL-1572^TM^), SK-OV-3 (ATCC^®^ HTB-77^TM^), APOCC (ovarian primary cell line derived from ascites fluid) (pers. communication Dr. Arash Tabrizi), A2780 (93112519-1VL, Sigma), A2780cis (93112519-1VL, Sigma), NIH: OVCAR-3 (ATCC^®^ HTB-161^TM^), lung cancer cell line—NCI-H226 (ATCC^®^ CRL-5826^TM^) and lung normal fibroblast cell line LL 24 (ATCC^®^ CCL-151^TM^), T lymphocyte cells Jurkat (ATCC number TIB-152^TM^) (all from American Type Culture Collection, Manassas, VA), were used for the current study. Human lymphoblastoid cell lines (LCL) derived from healthy (LCL-H2) and triple-negative breast cancer patients (LCL-TNBC) were isolated in Dr. Said Dermime’s lab (National Center for Cancer Care and Research, Hamad Medical Corporation, Doha, Qatar). Cells were cultured in DMEM or RPMI (Life Technologies, NY, United States) supplemented with 10% fetal bovine serum (Life Technologies, United States). Low passage number cells were used for all experiments. Cell culture was routinely checked for mycoplasma contamination using the MycoAlert Mycoplasma detection kit (Lonza, Basel, Switzerland). pcDNA3.1 plasmid and pcDNA3.1(+) CircRNA Mini Vector (Addgene) were used to carry out gain-of-function experiments.

### Silencing and Overexpression

Cell viability was checked using Trypan Blue and TC20^TM^ Automated Cell Counter (Bio-Rad). Cells were seeded at density (5 × 10^5^) cells/well in 6-well plates. For *NFATC3* silencing, cells were transfected after 24 h with 30 pmol concentration of siRNA (Integrated DNA Technologies) or scrambled control (Mission siRNA Universal Negative Control) using Lipofectamine^®^ RNAiMAX Reagent (Life Technologies) in Gibco^TM^ OptiMEM. siRNA transfection was carried out using two custom-designed siRNAs for both *NFATC3* circular and linear transcripts; sicircNFATC3-1, sicircNFATC3-2, silinNFATC3-1, silinNFATC3-2 and universal scrambled control and si-circular junction scrambled controls specific to circular RNA (Supplementary Table 1). For *NFATC3* overexpression, cells were transfected with 2.5 micrograms of each empty vectors and vectors containing *NFATC3* insert using Lipofectamine^®^ 3000 Transfection Kit (Life Technologies) and treated with G418 for more than 2 weeks to have a stable transfection.

### Overexpression Vector Preparation

pcDNA3.1(+) CircRNA Mini Vector (plasmid number 60648) (Liang and Wilusz, 2014) and pcDNA3.1 plasmid -both obtained from Addgene plasmid repository- were used for expressing *NFATC3* in cancer cells. circNFATC3 PCR product (Exon 2 and 3) was used for cloning templates. Cloning was conducted according to manufactures protocol using suitable restriction enzymes. The *NFATC3* construct in the pcDNA3.1(+) CircRNA Mini Vector containing *Alu* repeats (Liang and Wilusz, 2014) can potentially circularize the *NFATC3* construct spanning from Exon 2 to Exon 3. This is in contrast to its parental pcDNA3.1 Vector without *Alu* repeats which does not circularize exons thus both vectors were used for ectopic expression of exons 2 and 3 of *NFATC3* along with their respective empty vectors in MDA-MB-231 and SK-OV-3 cells. The *NFATC3* linear construct -exon 2 and exon 3- in pcDNA3.1 vector, its empty vector, and empty pcDNA3.1(+) CircRNA Mini Vector were used through the process of transfection as controls to identify the phenotypic effect of circNFATC3 in the overexpressed circNFATC3 cells.

### Nuclear or Cytoplasmic RNA Isolation

After 48 h transfection, RNA was isolated using SurePrep^TM^ Nuclear or Cytoplasmic RNA Purification Kit (Thermo Fisher Scientific), and the purity of nuclear and cytoplasmic extract was determined using nuclear and cytoplasmic gene-specific primers as described earlier (Karedath et al., 2019a).

### RNaseR Digestion

Total RNA was isolated using RNeasy Mini Kit (Qiagen) according to manufacturer’s protocols including on-column DNase digestion. For RNase R treatment, 2 micrograms of total RNA were briefly heated to 70°C to denature then cooled to 40°C on a thermocycler. 20 units of RNase R (Epicenter) and 1 unit/microliter Murine Ribonuclease Inhibitor (New England Biolabs) were added to the denatured RNA samples and incubated at 40°C for 1 h. Real-time PCR was performed for the RNA samples using gene or circRNA specific primers.

### NFATC3 Gene Expression

*NFATC3* circular and linear transcripts were investigated in 15 cell lines—MCF7, MDA-MB-231, SK-OV-3, LL 24, MCF 10A, NCI-H226, APOCC, A2780cis, A2780, OVCAR-3, PA-1, HMEC, LCL-TNBC, LCL-H2, Jurkat—using beta-actin and B2M as internal controls. Total RNA was isolated from whole cell lysate using miRNeasy Mini Kit (Qiagen) then quantified using Qubit RNA HS Assay Kit (Life technologies). cDNA synthesis was done using random primers for circRNA experiments from iScript^TM^ Select cDNA Synthesis Kit (Bio-Rad). Fast Start Universal SYBR Green fMaster Mix (Roche) was used to amplify the specific genes using cDNA primers obtained from Integrated DNA Technologies (IDT). Each Real-Time assay was done in triplicate using StepOnePlus Real-Time PCR System (Applied Biosystems) with various primer constructs (IDT) listed in Supplementary Table 1.

### RNA-Seq Analysis

After 48 h transfection, RNA isolation and DNase digestion using miRNeasy Mini Kit and RNase-Free DNase Set (Qiagen) was done to extract pure RNA. RNA quality control measurement was done using the High Sensitivity RNA Kit and RNA 6000 Nano Kit (Agilent Technologies). Ovation^®^ RNA-Seq System V2 (NuGEN) was used to prepare SPIA cDNA. Libraries were multiplexed using NEXTflex^TM^ DNA Barcodes (Biooscientific) for RNA-seq. RNA-seq library preparation, *in silico* detection of circRNA candidates and differentially regulated genes from paired-end RNA-seq data, was conducted as described earlier (Malek et al., 2012; Ahmed et al., 2016). Briefly, gene expression was estimated after aligning the RNA-seq data to the reference genome (GRCh38) in a paired-end aware manner using Tophat spliced read mapper and only keeping the concordant primary alignments. These were again filtered to remove any potential PCR duplicates with samtools rmdup. For each gene, expression was then quantified at the transcript level as the sum of paired-end fragments and excluding any chimeric fragments using the featureCounts package. Differential gene expression was estimated in three biological replicates of circNFTAC3 silenced MDA-MB-231 cells compared to control and identifying genes with at least twofold changes in expression at FDR of < 0.02 using PARTEK Genomic Suite^1^. Further analysis was performed with Metascape which is a web-based portal designed to provide a comprehensive gene list annotation and analysis resource for experimental biologist (Zhou et al., 2019). Statistically enriched terms (GO/KEGG terms, canonical pathways, hall mark gene sets), accumulative hypergeometric *p*-values and enrichment factors were calculated and used for filtering. Metascape applies a mature complex identification algorithm called MCODE to automatically extract protein complexes embedded in the large network. GO enrichment analysis was applied to each MCODE network to assign “meanings” to the network component. Visualizations of functional enrichment and interactome analysis results were thus extracted.

### Cell Proliferation Assay

Cells were seeded at density (5 × 10^3^) cells/well in flat-bottom 96-well plates. For *NFATC3* silencing, cells were transfected after 24 h with siRNAs for both *NFATC3* circular and linear transcripts then were incubated in 37°C CO_2_ injected incubator for (48, 72, 96, and 120 h). For *NFATC3* overexpression, cells were transfected after 24 h with empty vectors and vectors containing *NFATC3* insert. CellTiter 96^®^ AQueous One Solution Cell Proliferation Assay and CellTiter-Glo 3D Cell Viability Assay (Promega) were conducted according to the manufacturer’s instructions. Luminescence was measured by EnVision Multilabel Plate Reader (PerkinElmer).

### Cell Migration Assay

Cells were plated in rectangular cell culture plates using Cell Comb^TM^ Scratch Assay (Merck) and grown to 100% confluency. A wound was created using a cell comb then the medium was replaced with Gibco^TM^ OptiMEM, reduced serum medium, no phenol red. Cells were transfected with respective siRNAs and vectors as mentioned earlier. The distance between the two sides of the cell-free area was photographed using a 10X objective AXIO Zeiss epifluorescence microscope. The distance was measured using the Zeiss Zen Microscope software (Carl Zeiss Carpenteria, CA, United States).

### Trans-Well Migration and Invasion Assay

Cellular migration and invasion were determined using Corning^®^ Matrigel^®^ Invasion Chamber 6-Well Plate 8.0 Micron (Corning). 10% FBS DMEM was added to the lower chamber as a chemoattractant. Transfected cells were resuspended in serum-free DMEM, transferred to the upper chamber of the Matrigel, incubated for 72 h, then visualized under the microscope to count invading cells.

### 3D Model Experiments

3D anchorage-independent spheroids were developed in MDA-MB-231 cell lines by seeding the cells in ultra-low attachment 6-well plates (Corning) for 5 days to facilitate spheroid formation. Reverse transfection of the spheroids with siRNAs was conducted as described earlier (Karedath et al., 2019a).

### Cell Proliferation Assay in 3D Models

Cells were seeded at density (1 × 10^5^) cells/well in 96-well ultra-low attachment plates with Gibco^TM^ OptiMEM, reduced serum medium, no phenol red. Once spheroids were formed after 5 days, transfection was conducted with siRNAs. After 48 h of transfection, CellTiter 96^®^ AQueous One Solution Cell Proliferation Assay was conducted.

### Collagen Invasion Assay in 3D Model

copGFP Control Plasmid:sc-108083 (Santa Cruz Biotechnology) was used to express GFP in the cells. After 48 h of transfection, puromycin selection was done for the cells. copGFP transfected MDA-MB-231 cells were seeded in 6-well ultra-low attachment plates for 3D model formation. The cells were silenced using respective siRNAs then the 3D structures were embedded in the collagen matrix; Gibco^TM^ Collagen I, Rat Protein, Tail (Thermo Fisher Scientific) in Falcon^TM^ Chambered Cell Culture Slides (BD Falcon). The invasiveness was analyzed by visualizing the gel under a fluorescence microscope after 72 h.

### Assessment of Mitochondrial Function by Seahorse Extracellular Flux Analyzer

The Mitochondrial Oxygen Consumption Rate (OCR) and Extracellular Acidification Rate (ECAR) in MDA-MB-231 cells were assessed by Agilent Seahorse XF Cell Mito Stress Test Kit and Agilent Seahorse XF Cell Energy Phenotype Test Kit (Agilent Technologies) and run in Agilent Seahorse XFe96 Analyzer (Seahorse Bioscience) as per manufacturers protocol. Cells were seeded at density (5 × 10^3^) cells/well in Seahorse XF Cell Culture Microplate, transfected after 24 h and assay was completed 48 h after transfection.

### Statistical Analysis

Statistical analysis was performed using Student’s *t*-test or one-way analysis of variance and GraphPad Prism 5 software (GraphPad Software, San Diego, CA, United States). *P* < 0.05 was considered to indicate a statistically significant difference. The results presented as the mean ± SEM were analyzed by one-way ANOVA/two-way with Dunnett’s multiple comparisons test.

## Results

### Validation of CircNFATC3 in Cancer Cells

**FIGURE 1:**
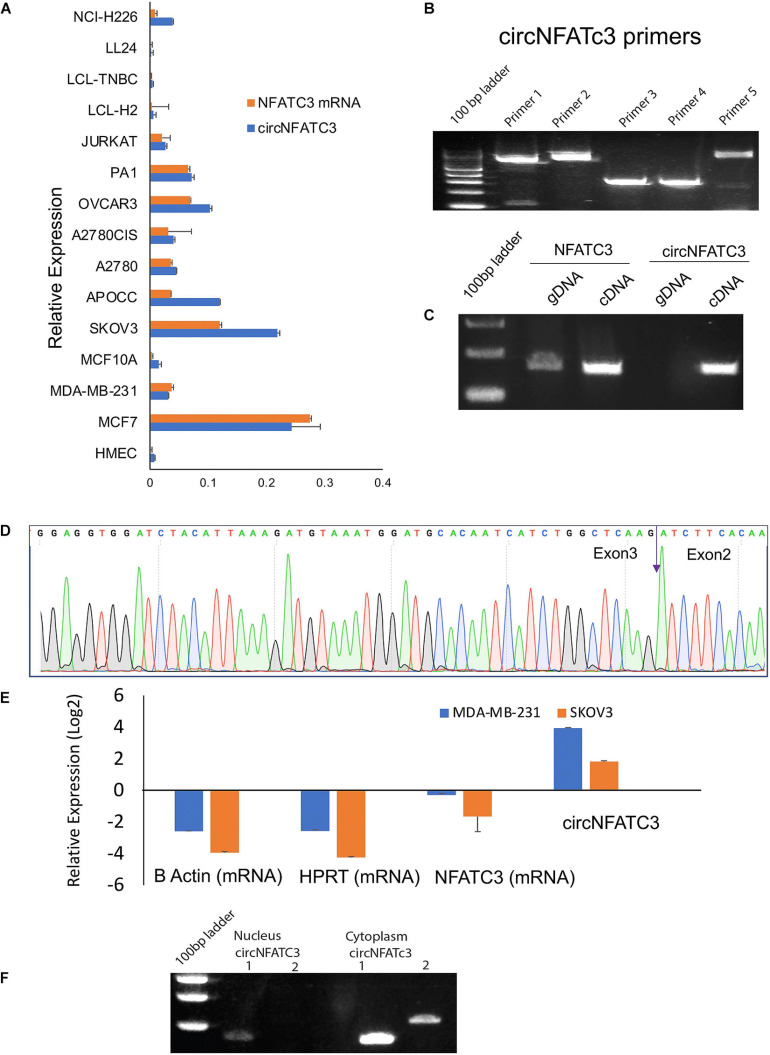
Validation of of circNFATC3 expression. **(A)** Real Time PCR shows abundance of NFATC3 isoforms in different cell lines. NFATC3 circular and linear transcripts were investigated in 15 cell lines using beta-actin and B2M as internal controls. **(B)** circNFATC3 backsplice junction amplification. Five different divergent primers were designed to amplify the backsplice exon junction. Each primer pair produced a single distinct band of expected PCR product size indicating the presence of the circular junction. **(C)** PCR amplification of NFATC3 DNA and cDNA using convergent primers. Convergent primers only amplify DNA samples, the cDNA samples amplifying both indicate the existence of circNFATC3. **(D)** Non-canonical circular splice junction (backsplice junction) of circNFATC3 exon 3-2. The backsplice junctional sequences were confirmed by Sanger sequencing. The backsplice junction is shown (blue arrow). **(E)** Real Time PCR analysis of RNase R resistance of circNFATC3. circRNAs are devoid of 3′ single strand overhangs, so they resist RNase R digestion. circNFATC3 is resistant to RNase R digestion compared to linear NFATC3, HPRT, and beta-actin in both cell lines; MDA-MB-231 and SK-OV-3. **(F)** Gel picture shows nuclear and cytoplasmic localization of circNFATC3. The PCR amplification of nuclear and cytoplasmic fractions of RNA demonstrate that circNFATC3 is predominantly localized in the cytoplasm, two primers were used to amplify the circular junctions.

Our previous studies reported that circNFATC3 is one of the most abundant circRNAs in cancer cells (Ahmed et al., 2016). A series of experiments were carried out to confirm circNFATC3 expression (Figure 1). We performed Real-Time quantitative PCR analysis on 15 different types of normal and cancer cell lines including breast, ovarian, lung, lymphoblastoid, to assess the cell-type-specific expression of circNFATC3 (Figure 1A). The majority of cancer cell lines showed an abundance of both *NFATC3* linear and circular transcripts. Some cancer cells expressed more circular RNA compared to linear parental mRNA. We further noticed that circNFATC3 derived from *NFATC3* gene exon 2 and exon 3 was abundant across different cancer cell lines compared to normal cells like HMEC, LL 24, and LCL cells. Breast and ovarian cancer cells showed a greater abundance of circNFATC3 compared to normal breast cell lines, lung fibroblast cells, and LCL cells. Five different divergent primers were designed to amplify the backsplice exon junction (Figure 1B). Each divergent primer pair produced a single distinct band of expected PCR product size indicating the presence of the circular junction (Figure 1B). The divergent primers -with respect to the genomic sequence- were used to validate circNFATC3 as they were only amplified for the cDNA template that was synthesized by random priming. While convergent primers only amplified the linear form on DNA sample, they showed amplification of both linear and circular forms on cDNA sample. This indicates that the circular RNA is a transcriptional splicing product rather than a form present in the genome (Figure 1C). The backsplice junctional sequences were confirmed by Sanger sequencing and the arrow indicates the backsplice junction between exon3 and exon2 (Figure 1D). As circRNAs are devoid of 3′ single strand overhangs, they are expected to show resistance to digestion by the exonuclease RNase R. We found that circNFATC3 is resistant to RNase R digestion compared to linear *NFATC3*, *HPRT1*, and beta-actin in MDA-MB-231 and SK-OV-3. Resistance to digestion with RNase R exonuclease confirms that circNFATC3 is a relatively stable circularized transcript (Figure 1E). The PCR analysis of nuclear and cytoplasmic fractions of RNA demonstrated that circNFATC3 is predominantly localized in the cytoplasm (Figure 1F). The purity of nuclear and cytoplasmic extraction is shown in Supplementary Figure 1.

### siRNA-Mediated Silencing of CircNFATC3 Is Highly Specific in Multiple Cancer Cells

**FIGURE 2:**
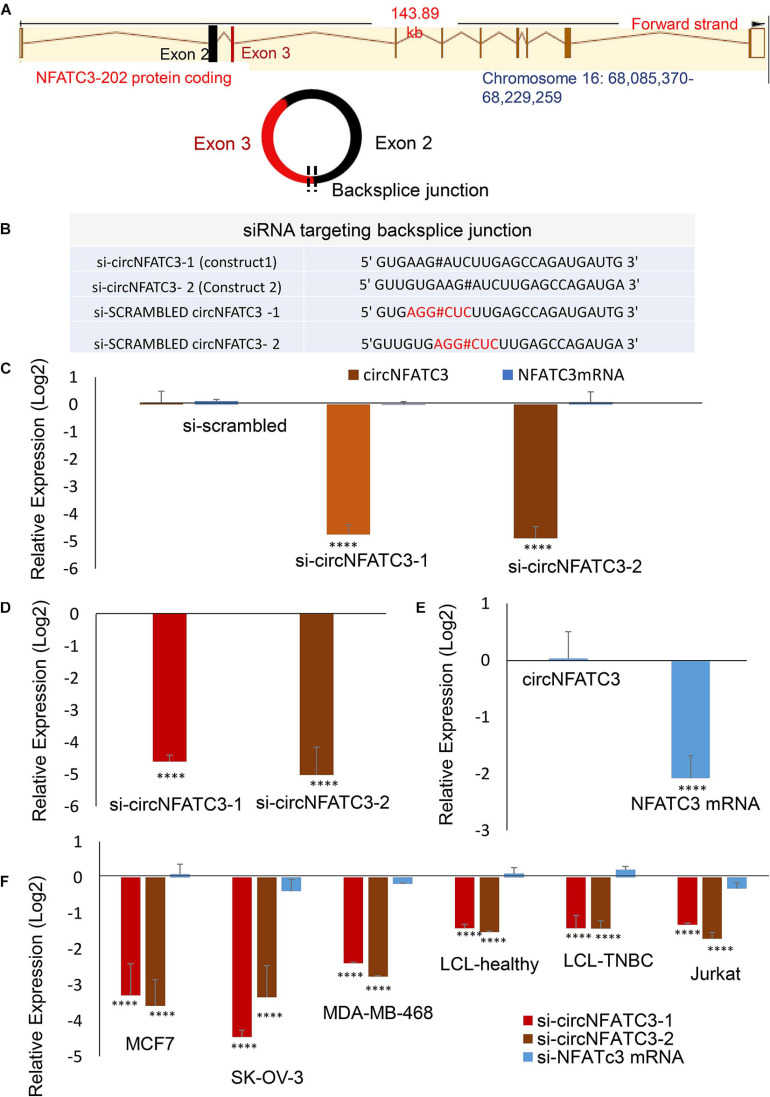
siRNA-mediated functional studies of circNFATC3 in cancer cells. **(A)** The location of NFATC3 in chromosome 16. The circNFATC3 is formed of exon 2 and 3 by backsplicing. **(B)** siRNA sequences targeting the backsplice junction of circNFATC3 and control siRNAs containing scrambled backsplice junction of circNFATC3. # representing the backsplice junction between exon3 and exon 2 of NFATC3. **(C)** Real Time PCR analysis of siRNA-mediated knockdown of circNFATC3. C: siRNAs were transfected into MDA-MB-231cancer cell lines to induce siRNA-mediated knockdown of the circular RNA keeping the linear RNA intact. Two different constructs of si-circNFATC3 were used, si-circNFATC3-1 and si-circNFATC3-2. **(D)** Knockdown efficiency (real time PCR) of the siRNA-mediated knockdown of circNFATC3 in MDA-MB-231 3D (three dimensional anchorage independent) model. **(E)** siRNA-mediated silencing of NFATC3 mRNA. Silencing NFATC3 mRNA using siRNAs targeting the linear NFATC3 (using two constructs silinNFATC3-1 and silinNFATC3- 2) preserved the circNFATC3 in MDA-MB-231 cell lines. **(F)** Real time PCR of siRNA-mediated silencing of circNFATC3 in different cell lines. Six cell lines that were transfected with two si-circNFATC3 constructs vs. scrambled siRNAs showed 65% to 95% knockdown efficiency of the circular junction. ****P < 0.0001. The *p*-value in **(C,E,F)** Was determined by two-way analysis ANOVA with Dunnett’s multiple comparisons test, the *p*-value in **(E)** Was determined by two-way analysis ANOVA with sidak’s multiple comparisons test. The *p*-value in **(D)** Was determined *t*-test (one tailed).

circNFATC3 originates from chromosome 16 with the backsplice junction forming between exons 2 and 3 (Figure 2A). To investigate the functional role of circNFATC3 in cancer cells, we custom designed siRNAs to target the backsplice junction and control siRNAs that have scrambled backsplice junction of circNFATC3 (Figures 2A,B). These siRNAs were transfected into MDA-MB-231 to induce siRNA-mediated knockdown of the circular RNA while keeping the linear RNA unaffected (Figure 2C). The circRNA-specific siRNA was designed targeting the backsplice junction spanning exons 2 and 3 of *NFATC3* gene. Three controls were used for the knockdown study, universal scrambled control and circNFATC3 specific scrambled controls as described in Figure 2B. MDA-MB-231 cells in an anchorage-independent 3D condition also showed a very high knockdown efficiency (Figure 2D). Silencing *NFATC3* mRNA using siRNA targeting the linear *NFATC3* showed knockdown of *NFATC3* mRNA retaining the circNFATC3 intact (Figure 2E). The circRNA knockdown specificity is demonstrated in Figure 2C and its parental gene knockdown using siRNA downstream of exon 3 and exon 2 (which is in exon 9) left the circRNA unaltered (Figure 2E). We used breast cancer cell lines MDA-MB-231 and MCF7 cells for further validation studies and experiments. However, we observed high knockdown efficiency ranging between 65 and 95% of the circular junction when using the respective siRNAs vs. the controls (scrambled siRNAs) in six cell lines’ transfections namely, MDA-MB-231, SK-OV-3, MCF7, MDA-MB-468, Lymphoblastoid Cell Line (LCL healthy, LCL TNBC) (Figure 2F). Using two siRNA constructs against the circNFATC3, we confirmed that the knockdown of circNFATC3 in MDA-MB-231 is specific and has no significant effect on the expression of the linear transcript of *NFATC3* (Figures 2C–E). These experiments clearly validate the specificity of siRNAs constructs as it targets only the circular RNA nor the linear counterpart of NFATC3.

### Silencing of CircNFATC3 Changes the Molecular Phenotypes of MDA-MB-231 Breast Cancer Cells

**FIGURE 3:**
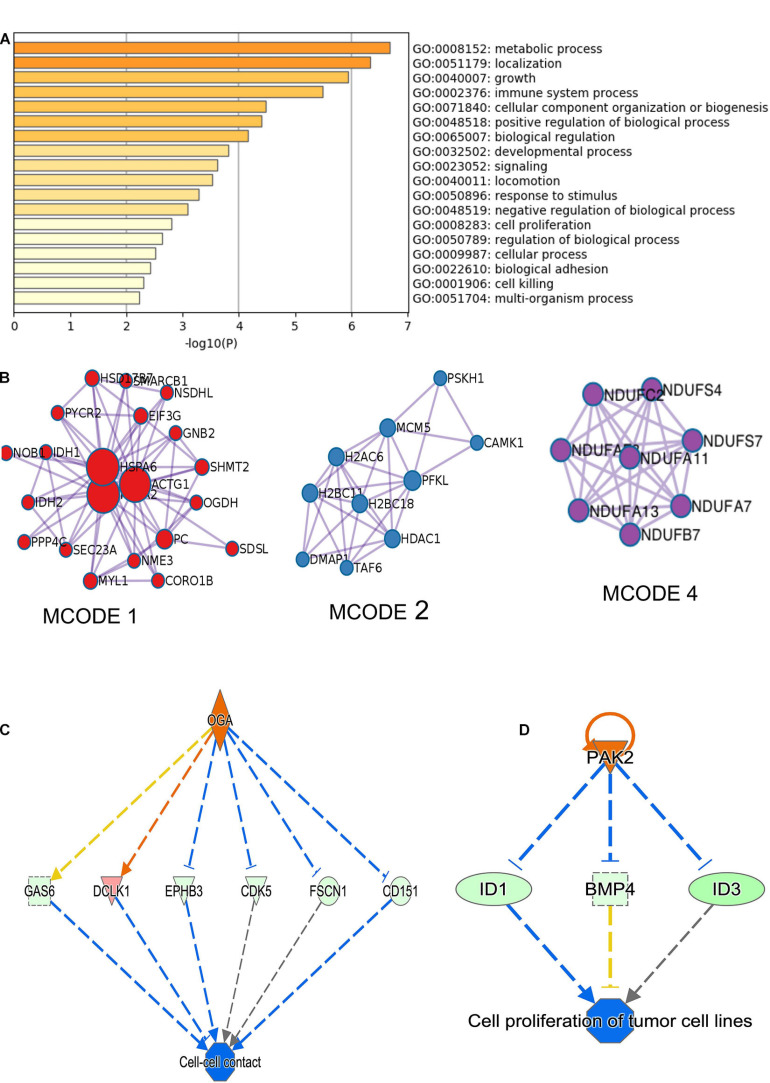
RNA-seq analysis of differentially expressed genes in circNFATC3 silenced MDA-MB-231 cells (circNFATC3 vs. control). **(A)** Metascape analysis of enriched ontology clusters were identified. **(B)** MCODE algorithm used to identify networks neighborhoods where proteins are densely connected. MCODE network analysis shows cluster of gene involved in gene function MCODE 1. Complex I biogenesis, cellular respiration, NADH dehydrogenase complex assembly, Biosynthesis of amino acids, Carbon metabolism, Citrate cycle (TCA cycle). MCODE 2. Chromatin organization, Chromatin modifying enzymes, HDACs deacetylate histones. MCODE 4. Complex I biogenesis, mitochondrial respiratory chain complex I assembly, NADH dehydrogenase complex assembly. The canonical pathways analysis by Ingenuity^®^ IPA toolkit identified enrichment of differentially regulated genes involved in many pathways including: **(C)** Cell-cell contact, **(D)** Networking analysis (IPA).

RNA-sequencing was performed in three biological replicates of the MDA-MB-231 cell line for both scrambled siRNA control and two siRNA constructs targeting circNFATC3. RNA-seq showed that 881 genes are differentially regulated in circNFATC3 knockdown cells compared to controls (837 upregulated and 43 downregulated). Differentially expressed genes in circNFATC3 knockdown cells with at least twofold (log2) change in expression and differentially regulated genes in circNFATC3 silenced MDA-MB-231 in comparison with control cells are listed in Supplementary File 1. Gene enrichment analysis and MCODE algorithm using Metascape tool (Zhou et al., 2019) were then applied to the differentially regulated genes to identify gene ontology clustering and identifying possible protein clusters. The neighborhoods where proteins are densely connected for enriched protein clusters revealed that gene involved in metabolic process, respiration, TCA cycle, mitochondrial functions were regulated (Figures 3A,B).

The canonical pathways analysis by Ingenuity IPA toolkit (IPA, QIAGEN Redwood City^2^, downregulation of genes involved in the cell-to-cell contact; Figure 3C and Supplementary Table 2). Network analysis of genes regulated by siRNA mediated knockdown of circNFATC3 compared to control in MDA-MB-231 cells shows deregulation of *STAT3* pathway, migration of endothelial cells, disruption in vasculogenesis by downregulation of *VEGFA*, synthesis of sterol by downregulation of *SIRT1* gene. The silencing of circNFATC3 regulates cell proliferation, migration, and inflammation and growth by silencing or activates cascade of genes involved in the pathway (Figure 3D and Supplementary Figure 9). The top canonical pathways that are regulated in circNFATC3 knockdown MDA-MB-231 cells compared to control are mitochondrial dysfunction, axonal guidance, pentose phosphate pathway, *PTEN* signaling, *IL15* production, *Sirtulin* signaling, and *NFkB* signaling (genes are listed in Supplementary File 1). We observed downregulation of *EGF*, *ID1*, *ID3*, and *AKT1* which regulate cell-to-cell contact and cellular movement. The silencing of circNFATC3 affects cellular bioenergetics by downregulating the key genes involved in oxidative phosphorylation and mitochondrial dysfunction (Table 1). Real-time quantitative PCR validation of RNA-seq data was done for *IDH2*, *ID1*, *KRT80*, and *CALCR* genes both in circNFATC3 silenced MDA-MB-231 breast cancer cells and SK-OV-3 ovarian cancer cells (Supplementary Figure 2). Transcriptome analysis of differentially expressed genes in circNFATC3 silenced breast cancer cell lines suggests a strong molecular phenotype thus we proceeded with the functional screening of circNFATC3 silenced cells using cell-based phenotypic assays in MDA-MB-231 and SK-OV-3.

**TABLE 1:** KEGG pathways showing differentially regulated pathways and genes involved in circNFATC3 silenced MDA-MB-231 cells.

**Pathways**	**Genes**	***P*-value**
Steroid biosynthesis	EBP, DHCR7, LSS, HSD17B7, NSDHL, DHCR24	8.14E-04
Metabolic pathways	ATP5D, NDST3, SGMS2, CNDP2, LSS, CHPF2, OGDH, ACOX3, NDUFS7, ST6GALNAC6, NDUFS4, ACOT8, SARDH, DHCR24, PDXK, ACO2, PFKL, ACADS, ALDH5A1, NDUFC2, PDXP, NDUFA13, MOGS, PIGQ, NDUFA11, ATP6V1F, PYCR2, PGLS, MTMR14, DHRS4, NME3, G6PD, H6PD, MVK, MGAT5B, BCAT2, NDUFB7, POLR2L, MVD, GALNT4, G6PC3, PLPP2, JMJD7-PLA2G4B, DHCR7, PEMT, PAFAH1B3, FASN, IDH2, IDH1, UCK1, TSTA3, HSD17B7, NSDHL, EBP, SHMT2, A4GALT, COX8A, NDUFA7, SDSL, TKT, DBH, MMAB, GAPDHS, DPM2, DPM3, MAT2B, NDUFC2-KCTD14, PC	0.001278828
Rap1 signaling pathway	FGFR4, FGFR3, MAP2K2, BCAR1, CSF1, SIPA1, FPR1, RGS14, AKT1, ACTG1, ID1, CNR1, RRAS, PDGFRB, NGFR, EGF, MAP2K6, CSF1R	0.003165669
Axon guidance	PLXNA1, LIMK1, PLXNB2, PLXNB3, DPYSL2, EPHB3, CDK5, SEMA6B, RGS3, PAK4, RHOD, SEMA4D	0.003681465
Pentose phosphate pathway	PGLS, G6PD, PFKL, H6PD, TKT	0.024544865
Oxocarboxylic acid metabolism	BCAT2, ACO2, IDH2, IDH1	0.026263637
PI3K-Akt signaling pathway	CRTC2, FGFR4, PHLPP2, TNXB, FGFR3, MAP2K2, CSF1, PKN1, BAD, G6PC3, BCL2L11, AKT1, CASP9, GNB2, ITGB7, ATF6B, TSC2, COL6A2, PDGFRB, NGFR, EGF, CSF1R	0.026486479
Citrate cycle (TCA cycle)	ACO2, IDH2, IDH1, OGDH, PC	0.027482744
Oxidative phosphorylation	ATP5D, NDUFS7, NDUFS4, NDUFB7, NDUFA7, COX8A, NDUFC2, NDUFA13, NDUFC2-KCTD14, ATP6V1F, NDUFA11	0.033556828
Ras signaling pathway	FGFR4, FGFR3, MAP2K2, CSF1, BAD, MAPK10, AKT1, JMJD7-PLA2G4B, GNB2, PAK4, RRAS, PDGFRB, NGFR, EGF, CSF1R	0.056040211
Regulation of actin cytoskeleton	FGFR4, FGFR3, LIMK1, MAP2K2, BCAR1, PPP1R12C, MYL9, ACTG1, PAK4, ITGB7, RRAS, PDGFRB, BRK1, EGF	0.064361279

### siRNA-Mediated Silencing of CircNFATC3 Decreases Cell Proliferation, Migration, and Invasion in Breast and Ovarian Cancer Cells

**FIGURE 4:**
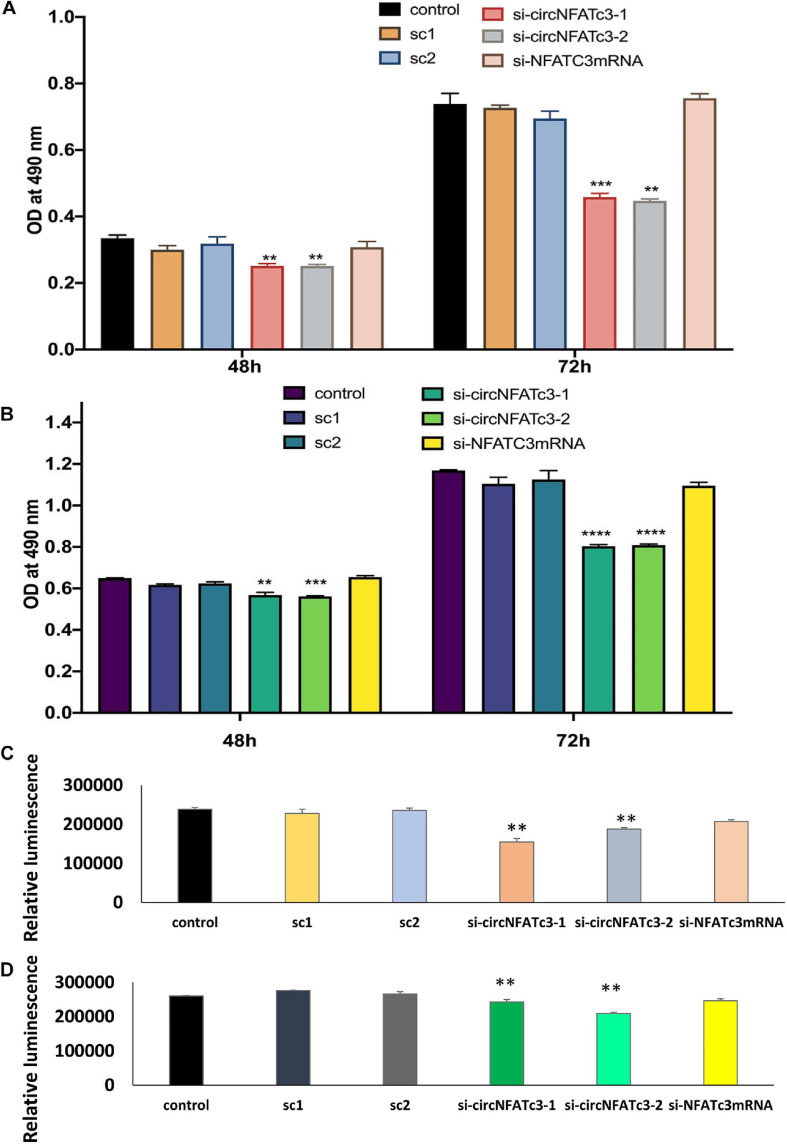
Cell proliferation (MTS and ATP) assays in circNFATC3 and NFATc3 mRNA silenced cells. The cell proliferation assays indicate that silencing of NFATC3 parental gene can induce a significant reduction in cell viability but not as substantial as its circular counterpart. **(A)** MTS assay of NFATC3 silenced MDA-MB-231 cells. **(B)** MTS assay of NFATC3 silenced SK-OV-3. **(C)** ATP assay of NFATC3 silenced MDA-MB-231 cells. **(D)** ATP assay of NFATC3 silenced SK-OV-3 cells. Sc1 and Sc2 are abbreviation for scrambled control 1 and scrambled control 2 of circNFATC3 respectively, whereas control is a universal siRNA control. Data in **(A–D)** are the means with error bars indicating standard error of the mean (SEM) of three experiments/biological replicates, **P < 0.01, ***P < 0.0005, ****P < 0.0001. The *p*-value in **(A,B)** was determined by two-way analysis ANOVA with Dunnett’s multiple comparisons test, the *p*-value in **(C,D)** is determined by one-way analysis ANOVA with Dunnett’s multiple comparisons test.

MTS cell proliferation and ATP assays showed a significant reduction in cell proliferation in circNFATC3 silenced cells compared to cells transfected with the scrambled siRNA controls (Figure 4). This reduction in both MTS and ATP cell proliferation assays was seen in MDA-MB-231 as well as SK-OV-3 cells (Figures 4A–D). The cell proliferation assays -MTS and ATP- indicate that the silencing of the *NFATC3* parental gene can induce a significant reduction in cell viability but not as substantial as its circular counterpart. Both cell lines showed a significant reduction in cell proliferation after 72 h of transfection, however, MDA-MB-231 cells were able to show a reduction in cell proliferation as early as 48 h of transfection (Figure 4A). These results suggest that knockdown of circular forms of *NFATC3* is capable of inducing strong phenotypic changes and modulating the growth of cancer cells.

**FIGURE 5:**
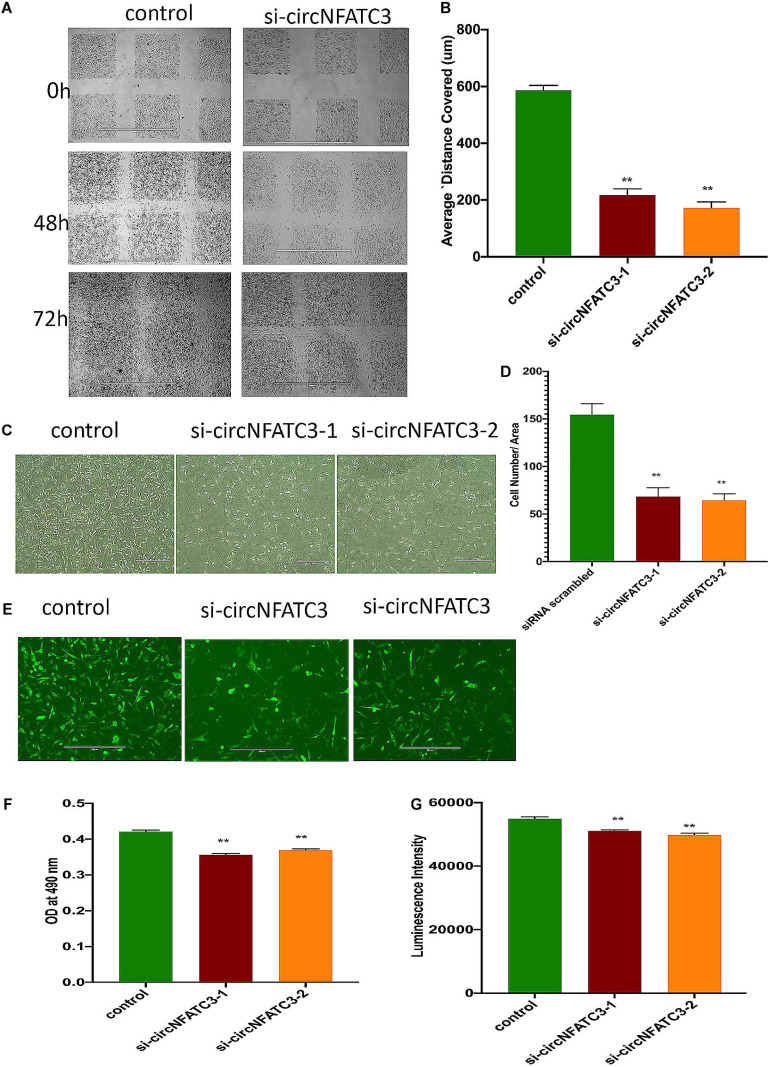
Migration and invasion assays of circNFATC3 silenced MDA-MB-231 cells. **(A)** wound healing assays. circNFATC3 silenced cells reduce migration after 48 and 72 h compared to control. **(B)** Scratch distance covered average. Wound healing analysis shows a significant reduction in migration of circNFATC3 silenced cells compared to control cells. **(C)** Boyden chamber. It shows a significant reduction in the invasion and migration of circNFATC3 silenced cells compared to control cells at 72 h. **(D)** circNFATC3 silenced MDA-MB-231 cells and control cells penetration through matrigel at 72 h. circNFATC3 silenced cells number that traveled in the denuded space is less than the number of the control cells. **(E)** Collagen invasion assay of circNFATC3 silenced cells in the 3D model. The circNFATC3 silenced cells exhibit less invasion fail dispersion through the collagen matrix compared to control MDA-MB-231 cells at 72 h. **(F,G)** represents Cell proliferation (MTS and ATP) assays in circNFATC3 in 3D phenotype of MDA-MB-231 cells. Data in **(B,D,F,G)** are the means with error bars indicating standard error of the mean (SEM) of three experiments. **P < 0.01. The *p*-value in **(B,D,F,G)** is determined by one-way analysis ANOVA with Dunnett’s multiple comparisons test.

The wound-healing assay showed that the silencing of circNFATC3 reduces cell migration in MDA-MB-231 (Figures 5A,B) after 48 and 72 h compared to the scrambled control. Matrigel invasion -inserts coated with matrigel- analysis using the Boyden chamber showed that circNFATC3 silenced cells undergo a significant reduction in invasion and migration compared to the scrambled control at 72 h time point (Figures 5C,D). The collagen invasion assay of the circNFATC3 silenced cells in 3D anchorage-independent conditions exhibited less invasion and failed dispersion through the collagen matrix compared to scrambled control (Figure 5E). The 3D anchorage-independent model of MDA-MB-231 cells showed the same phenotypic effects -reduction in cell proliferation- like 2D condition (Figures 5F,G).

### siRNA-Mediated Silencing of circNFATC3 Modulates Cellular Bioenergetics Showing a Shift in Metabolic Phenotype

**FIGURE 6:**
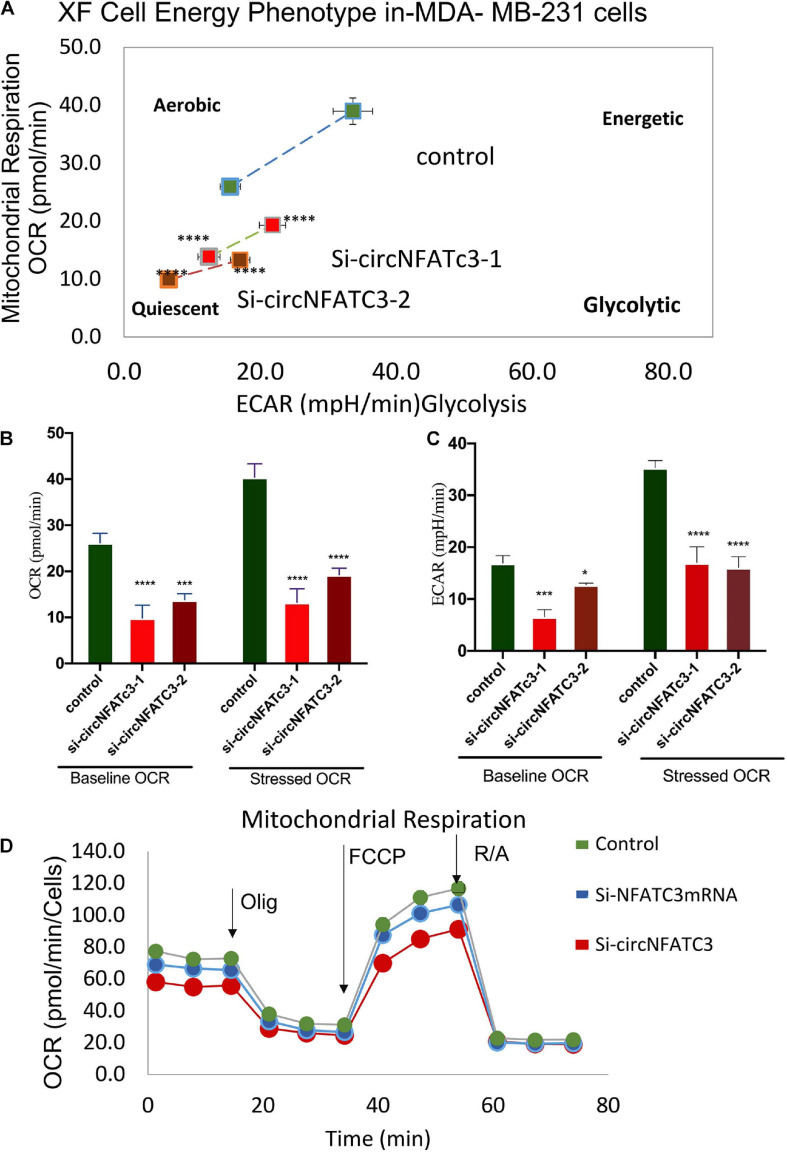
Metabolic alteration in circNFATC3 and NFATC3 mRNA silenced MDA-MB-231 cells. **(A)** XF cell energy phenotype in circNFATC3 silenced cells. circNFATC3 silenced cells maintain a quiescent metabolic phenotype with low OCR and ECAR compared to control cells. **(B)** Oxygen consumption rate in circNFATC3 silenced cells. OCR is significantly low in circNFATC3 silenced cells compared to control cells. **(C)** Extracellular acidification rate in circNFATC3 silenced cells. ECAR is significantly low in circNFATC3 silenced cells compared to control cells. **(D)** Mitochondrial respiration of the circNFATC3 silenced cells, Olig is (Oligomycin); FCCP [carbonylcyanide-p-(trifluoromethoxy) phenylhydrazone] and R/A (Rotenone/Antimycin). Data in **(B,C)** are the means with error bars indicating standard error of the mean (SEM) of three experiments. *P < 0.02, ***P = 0.0005, ****P < 0.0001. The *p*-value in **(A,B)** was determined by two-way analysis ANOVA with Dunnett’s multiple comparisons test.

RNA-seq analysis revealed that knockdown of circNFATC3 can regulate oxidative phosphorylation and TCA cycle (Tricarboxylic Acid Cycle), thereby affecting the mitochondrial function directly. We used extracellular flux assays which allow direct evaluation of cellular bioenergetic profiles *ex vivo* by measuring oxygen consumption rate (OCR, a measure of oxidative phosphorylation) and extracellular acidification rate (ECAR) and cell energy phenotype. To functionally validate the RNA-seq results, we performed extracellular flux analysis for mitochondrial potential and cell energy phenotype in MDA-MB-231 and SK-OV-3 cells (Figure 6A and Supplementary Figure 3A). As per the RNA-seq data, we found that both the respiratory capacity and aerobic glycolysis, as measured by OCR and ECAR, respectively, of circNFATC3 silenced cells were significantly lower compared to the scrambled controls (Figures 6A–D). Figures 6B,C represent two different types of cell energy phenotypes; (a) the baseline which is OCR and ECAR of cells at starting assay conditions (specifically in the presence of a non-limiting quantity of substrates) and (b) the stressed phenotype which is OCR and ECAR of cells under an induced energy demand (specifically in the presence of stressor compounds). These results indicate that circNFATC3 knocked down cells exist in a relatively low bioenergetic state while scrambled control cells adapt an energetic (i.e., high respiratory capacity, high glycolysis) metabolic phenotype (Figure 6A). SK-OV-3 cells also follow the same trend, as silencing circNFATC3 lowers mitochondrial respiration (Supplementary Figure 3A). Taken together, these data show that knocking down circNFATC3 in cancer cells can maintain a quiescent metabolic phenotype demonstrating low respiratory capacity and glycolysis compared to control cells.

### Overexpression of CircNFATC3 Regulates Cell Proliferation, Migration and Cellular Bioenergetics

**FIGURE 7:**
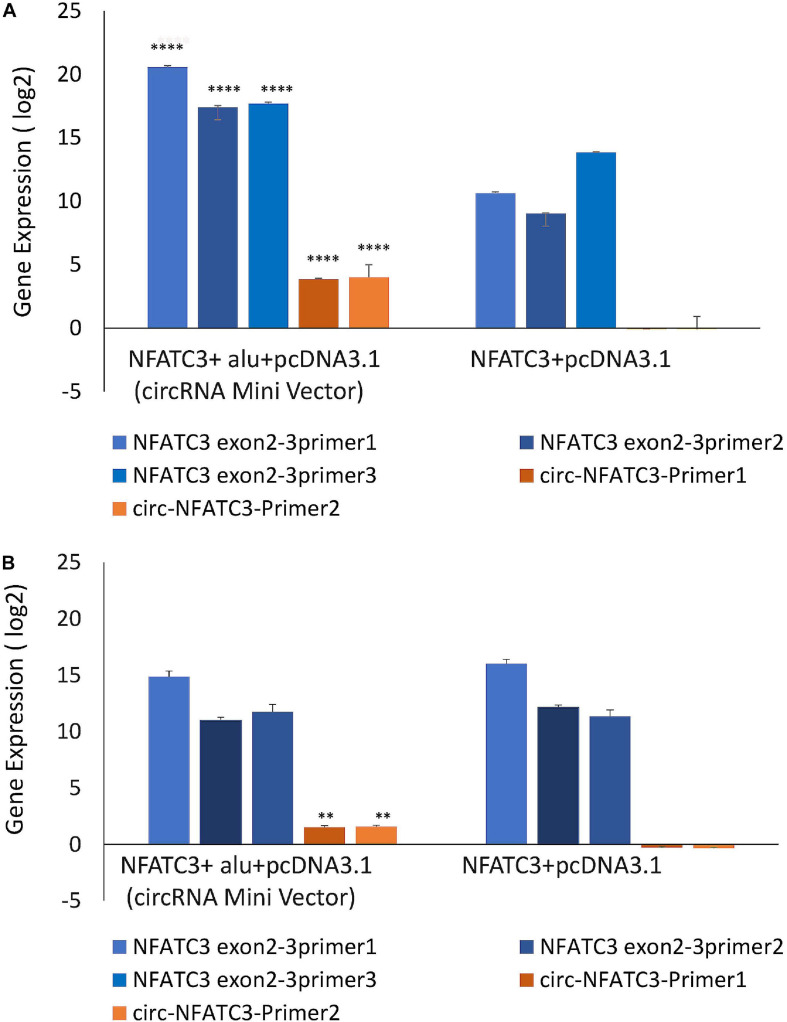
Gain-of-function assay of the NFATC3 gene using pcDNA3.1(+) CircRNA Mini Vector and pcDNA3.1 Vector using Real time PCR analysis (relative gene expression compared to its respective control vector). NFATC3 construct in the pcDNA3.1(+) CircRNA Mini Vector expressed both circNFATC3 and NFATC3 mRNA, but the pcDNA3.1 Vector was not able to circularize the NFATC3 construct. (A) NFATC3 overexpression in MDA-MB-231 cells. (B) NFATC3 overexpression in SK-OV-3 cells. **P < 0.01, ****P < 0.0001. The *p*-value in (A,B) was determined by two-way analysis ANOVA with Sidak’s multiple comparisons test.

MDA-MB-231 and SK-OV-3 cells were used for gain-of-function assays due to their moderately high expression of circNFATC3 and high level of transfection efficiency. MDA-MB-231 cells showed high transfection efficiency and were able to overexpress 16-fold higher levels of circularized *NFATC3* construct compared to empty vector with *Alu* repeats (circRNA Mini Vector). We ectopically overexpressed circNFATC3 in MDA-MB-231 and SK-OV-3 cells using pcDNA3.1(+) CircRNA Mini Vector. For comparison, we also overexpressed the linear form of exon 2 and 3 using the pcDNA3.1 original vector that does not allow subsequent circularization of the structure due to its lack of *Alu* repeats (Figures 7A,B). The *NFATC3* construct which is composed of exon 2 and exon 3 is 1,298 bp long (cloning details are shown in Supplementary Figure 8). Only the *NFATC3* construct in pcDNA3.1(+) CircRNA Mini Vector with *Alu* repeats was able to circularize the majority of the transcript in MDA-MB-231 and SK-OV-3 cells (Figures 7A,B).

**FIGURE 8:**
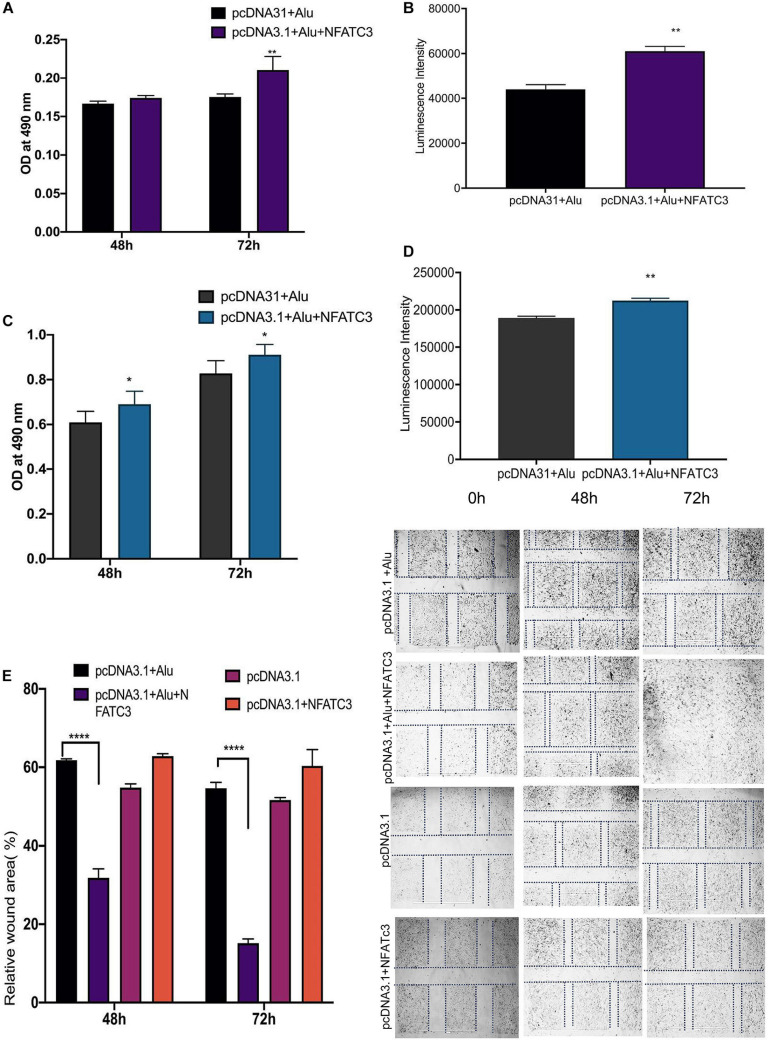
Phenotypic assays of the NFATC3 overexpressed cells. **(A)** MTS assay of the NFATC3 overexpressed MDA-MB-231 cells. **(B)** ATP assay of the NFATC3 overexpressed MDA-MB-231 cells. **(C)** MTS assay of the NFATC3 overexpressed SK-OV-3 cells. **(D)** ATP assay of the NFATC3 overexpressed SK-OV-3 cells. **(E)** Scratch covered distance average measured at 0, 48, and 72 h in MDA-MB-231 cells. Wound healing analysis shows a significant increase in migration of the circNFATC3 overexpressed cells compared to control cells. **(F)** Wound healing assay. circNFATC3 overexpressed cells increase migration after 48 and 72 h relative to 0 h. Data in **(A–E)** are the means with error bars indicating standard error of the mean (SEM) of three experiments. *P < 0.04, **P = 0.01, ****P < 0.0001. The *P*-value in **(A,B)** was determined by two-way analysis ANOVA with Dunnett’s multiple comparisons test.

**FIGURE 9:**
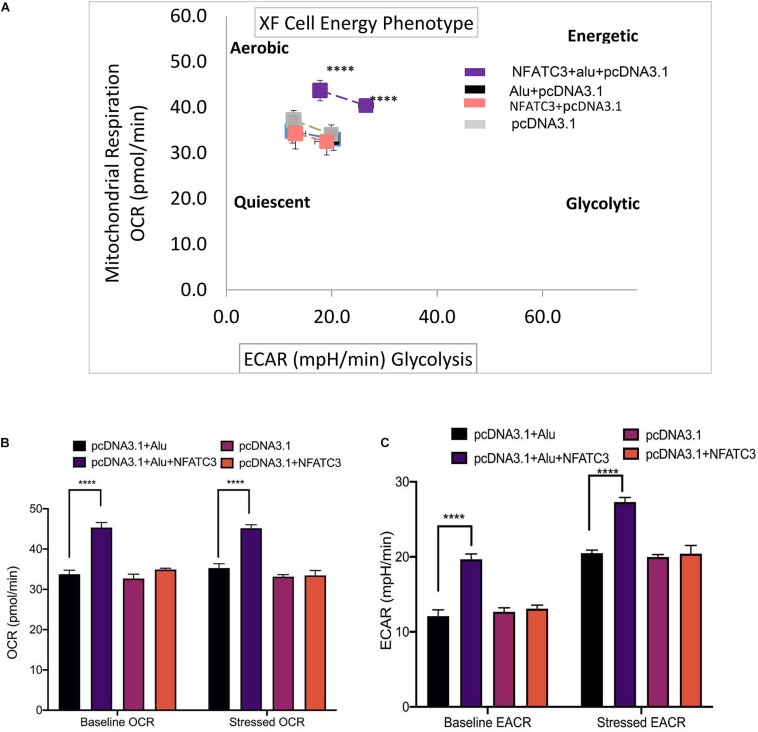
Metabolic alteration in circNFATC3 and NFATC3 mRNA overexpressed MDA-MB-231 cells. **(A)** XF cell energy phenotype in circNFATC3 overexpressed cells. circNFATC3 overexpressed cells maintain an aerobic metabolic phenotype with high OCR and ECAR compared to control cells. **(B)** Oxygen consumption rate in circNFATC3 overexpressed cells. OCR is high in circNFATC3 overexpressed cells compared to control cells. **(C)** Extracellular acidification rate in circNFATC3 overexpressed cells. ECAR is significantly high in circNFATC3 overexpressed cells compared to control cells. Data in **(B,C)** are the means with error bars indicating standard error of the mean (SEM) of three experiments. ****P < 0.0001. The *P*-value in **(A,B)** was determined by two-way analysis ANOVA with Dunnett’s multiple comparisons test.

Our next aim was to identify the phenotypic effect of the circularized transcript compared to the non-circularized control transcript. Cell proliferation assays at 48 and 72 h revealed increases in both cell lines of the circNFATC3 overexpressed groups compared to controls (Figures 8A–D). Moreover, overexpression of circNFATC3 dramatically enhanced cell migration in MDA-MB-231 cells (Figures 8E,F). We further determined the alteration of cellular bioenergetics of the overexpressed circNFATC3 and control in MDA-MB-231 and SK-OV-3 cells by using extracellular flux analysis (Figure 9 and Supplementary Figures 3B, 4). As shown in Figure 9, a significant increase was observed in OCR and ECAR with a slight shift in energy phenotype only in circNFATC3 overexpressed cells compared to all other control conditions (Figures 9A–C). Taken together, the overexpression of circNFATC3 can increase cancer cell proliferation, migration, and bioenergetics.

### Interactome Analysis CircNFATC3 Shows Association With hsa let7a-5p and Several RBP Binding Sites

**FIGURE 10:**
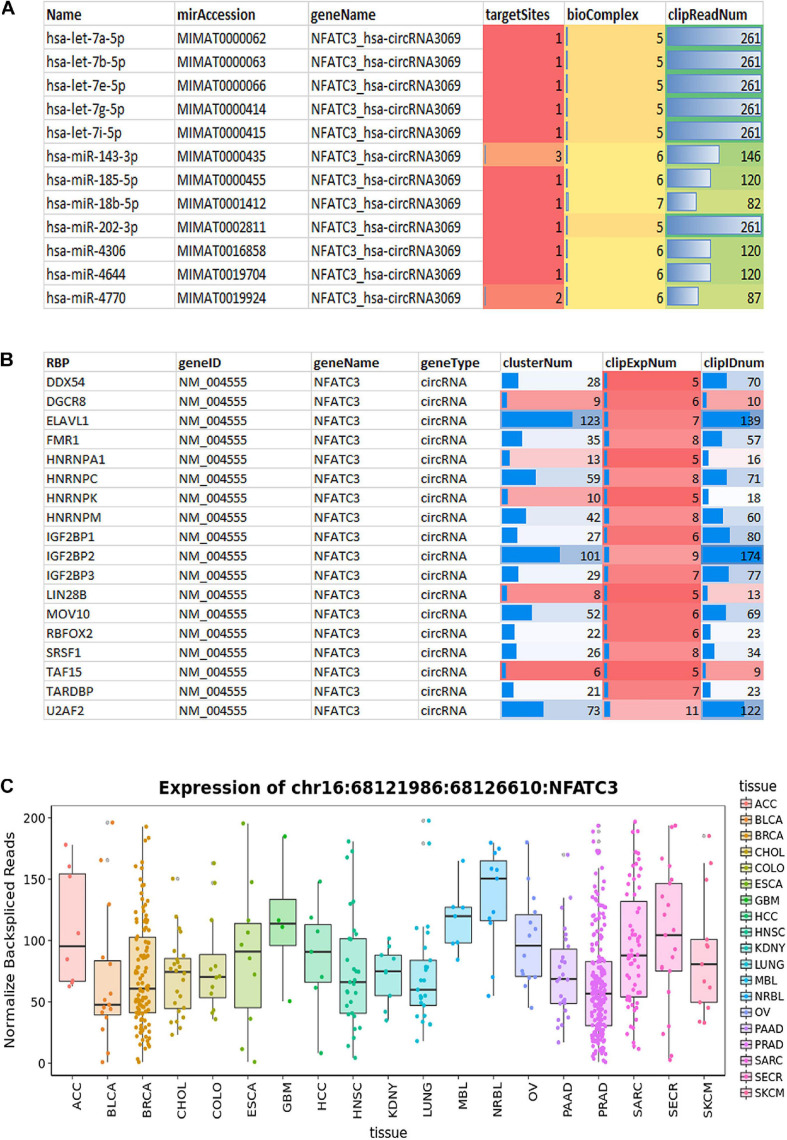
**(A)** StarBase v2.0 (http://starbase.sysu.edu.cn/) used to systematically identify the circNFATC3–miRNA and circNFATC3–RBP interaction networks from CLIP-Seq (PAR-CLIP, HITS-CLIP, iCLIP, CLASH) data sets generated by various independent studies. **(A)** StarBase analysis of circNFATC3 association with microRNAs. **(B)** Analysis of RBPs and its association with circNFATC3. **(C)** MiOncocirc analysis detect circNFACT3 isoform as one of the prominent circular RNA isoform in RNA seq data of Castration-Resistant Prostate Cancer, Pediatrics Tumors, Advanced Metastatic Cancers.

We characterized and identified circNFATC3 as an exonic circRNA consisting of two exons with a length of 1,298 bp that is resistant to RNase R treatment (Figure 1E). The annotated circNFATC3 isoforms listed in circnet^3^ (Liu et al., 2016) share the same backsplice junction studied here and are highly expressed in different tissues; particularly in breast cancer tissue compared to normal tissue (Supplementary Figure 5). circNFATC3 is annotated as hsa_circ_0000711 (circBase)^4^, NFATC3_hsa-circRNA3069 (starBase^5^, and chr16:68121986:68126610:NFATC3 by MiOncoCirc^6^. circNFATC3 is associated with tumor suppressor micro RNAs, let7-5p, and MiR-143-3p, and which is identified by publicly available circRNA data base (Figure 10A). CircNFATC3 is associated with RBP-RNA interactions which is supported by the identification of binding sites of RBPs derived from ChIP-seq data (starBase). Many known RBPs are associated with circNFATC3 indicates the regulatory and functional potential circular NFATC3 in cancer and other diseases (Figure 10B and Supplementary Figure 7). Moreover, circNFATC3 one of the most abundant circular isoform present in different tissues as identified by MiOncoCirc data base which is a compendium of circular RNAs compiled from cancer clinical samples at The University of Michigan (Figure 10C). Briefly, circNFATC3 is one of the moderately abundant and functionally active circular RNA in different human tissues.

## Discussion

Due to the extensive use of high−throughput sequencing platforms to identify novel regulatory RNAs, increasing numbers of circRNAs have been identified in human samples. The emerging evidence demonstrating that circRNAs play crucial roles in carcinogenesis and cancer progression have led to a rapid exploration of the functional relevance of these RNAs in cancer. Similar to oncogenes, aberrantly expressed circRNAs have been reported in diverse cancer types (Huang et al., 2017; Yang et al., 2018; Zeng et al., 2018). We identified circNFATC3 which is a highly expressed circRNA in breast and ovarian cancer cells (Ahmed et al., 2016). circNFATC3 has been identified as a potential biomarker in CRC cancer as it is highly expressed in myotonic dystrophy and brain cells regulating aging (Gruner et al., 2016; Li et al., 2018; Czubak et al., 2019). Even though circNFATC3 is annotated by different circRNA based databases, it remains functionally uncharacterized. The only documented functional characterization of circNFATC3 is its association with RNA binding protein *IMP3* which is potentially involved in the biogenesis of circular RNAs (Schneider et al., 2016).

As a step toward functionally characterizing the circNFATC3 of exon 2 and 3, we conducted circRNA silencing without altering the expression of its parental mRNA. A series of experiments were conducted to validate the knockdown efficacy in different cell lines using two siRNA constructs, a universal scrambled control, and two scrambled constructs of circNFATC3 (Figure 2C). Knockdown of circNFATC3 in MDA-MB-231 and SK-OV-3 cells that have a moderately high level of circNFATC3 showed a reduction in cell proliferation. However, knockdown of circNFATC3 in LCL (lymphoblastoid) cells which have a low level of circNFATC3 expression was unable to induce any phenotypic changes (Supplementary Figure 6 and Figure 1A). The presence of circNFATC3 was analyzed in different tissue types using MiOncoCirc database (Figure 10C). These results suggest that circNFATC3 knockdown is highly specific to circular RNA and has no off-target effect. Transcriptome analysis of circNFATC3 silenced MDA-MB-231 cells compared to scrambled cells shows a distinct molecular phenotype (Figure 3). The RNA-seq data for circNFATC3 silenced cancer cells shows a predicted decrease in the activity of migration, invasion, and mobility as the molecules involved in these pathways are affected which is consistent with our results of the migration and invasion assays for circNFATC3 silenced cells. circNFATC3 silenced cancer cells are predicted to decrease lipid synthesis, cell-to-cell contact and angiogenesis thus may cause cell cycle arrest which is evident in the phenotypic changes of circNFATC3 silenced MDA-MB-231 and SK-OV-3 cells. Cell proliferation, cell-to-cell contact, cell movement, and oxidative phosphorylation are altered in circNFATC3 silenced MDA-MB-231 cells (Figure 3 and Table 1). Cell proliferation assay revealed that knockdown of circNFATC3 can significantly reduce cell proliferation, cell migration, and cancer invasion (Figures 4, 5). circRNA junction -backsplice junction- is essential for altering the phenotype as evidenced by the circNFATC3 silencing and overexpression studies (Figures 2, 7) compared to the linear *NFATC3* knockdown. The scrambled siRNA with altered junctional sequence (Figure 2B) and pcDNA3.1 vector without *Alu* repeats that did not circularize the linear construct fail to show any phenotype in loss-of-function and gain-of-function studies, respectively. circNFATC3 plays an essential role in oxidative phosphorylation in MDA-MB-231 and SK-OV-3 cells as evidenced by extracellular flux analysis measuring mitochondrial stress and cell energy phenotype (Figures 6, 9).

Emerging evidence suggests that circRNAs are involved in complicated functions such as acting as endogenous RNAs to sponge miRNAs, protein decoys, protein translation, regulating the expression of parental genes, modulating alternative splicing, regulating RNA–protein interactions, and acting as scaffolds in the assembly of protein complexes (Ashwal-Fluss et al., 2014; Du et al., 2016; Liang et al., 2019; Su et al., 2019). Interactome analysis of circNFATC3 interaction with miRNA-circRNA networking using starBase v3.0 (Li et al., 2014) shows that circNFATC3 is highly associated with Let-7 family members of microRNAs (Figure 10A). The Let-7 microRNA family exerts its tumor suppressor and antiproliferative activities by repressing several oncogenes including *RAS* and by controlling key regulators of the cell cycle, cell differentiation, and apoptotic pathways (Johnson et al., 2005; Barh et al., 2010). Let-7 microRNA is known to be involved in a negative feedback loop that downregulates *NFAT* family gene expression (Kannambath, 2016). Similar to its parental gene family, circNFATC3 may also be involved in regulating gene expression *via* the Let-7-mediated feedback loop. Using StarBase to find RBP-circRNA interactions supported by CLIP-Seq data shows circNFATC3 association with several RBPs -RNA Binding Proteins- (Figure 10B). Likewise, using the circRNA interactome (Dudekula et al., 2016)^7^ tool shows an association between several RBPs and circNFATC3 (Supplementary Figure 7). As evidenced by circBase and circinteractome, circNFATC3 can potentially sponge microRNAs and RBPs making it important in regulating various biological activities including cell proliferation, motility, apoptosis, senescence, and cell responses to oxidative stress via posttranscriptional regulation such as RNA alternative splicing, conservation, transport and translation (Barh et al., 2010; Kannambath, 2016).

## Conclusion

To conclude, circNFATC3 is one of the uncharacterized circular RNA which holds a potential therapeutic agent. circNFATC3 is involved in regulating cell proliferation, cancer cell invasion, migration, and oxidative phosphorylation which highlights its important role in cancer progression.

## Data Availability Statement

The datasets presented in this study can be found in online repositories. The names of the repository/repositories and accession number(s) can be found below: NCBI BioProject, accession no: PRJNA680757 (https://www.ncbi.nlm.nih.gov/bioproject/?term=680757).

## Author Contributions

JM: conceptualization, funding acquisition, project administration, and supervision. TK, FA-D, IA, AA-Q, SA, AA, YA, and SD: data curation. TK and IK: formal analysis. TK and JM: investigation and visualization. TK, FA-D, AA-Q, AR, SA, AA, YA, SD, and JM: methodology. AR, AA, YA, SD, and JM: resources. TK, IK, AA, YA, SD, and JM: software. TK, FA-D, AA, YA, SD, and JM: validation. TK and FA-A: writing—original draft. SA and JM: writing—review and editing. All authors contributed to the article and approved the submitted version.

## Conflict of Interest

The authors declare that the research was conducted in the absence of any commercial or financial relationships that could be construed as a potential conflict of interest.

## Footnotes

1 https://www.partek.com/partek-genomics-suite/

2 https://www.qiagenbioinformatics.com/products/ingenuity-pathway-analysis/reveals

3 http://circnet.mbc.nctu.edu.tw/

4 http://www.circbase.org/cgi-bin/webBlat

5 http://starbase.sysu.edu.cn/index.php

6 http://ec2-52-91-79-148.compute-1.amazonaws.com:3838/circRNA

7 https://circinteractome.nia.nih.gov/
