# Supplementary material for: Regulation of Circular RNA CircNFATC3 in Cancer Cells Alters Proliferation, Migration, and Oxidative Phosphorylation
Source: Front Cell Dev Biol. 2021 Mar 19;9:595156. doi: 10.3389/fcell.2021.595156 (PMC8017239; doi:10.3389/fcell.2021.595156)
Supplement: Supplementary file 2 [file Presentation_1.PPTX]

## Slide 1
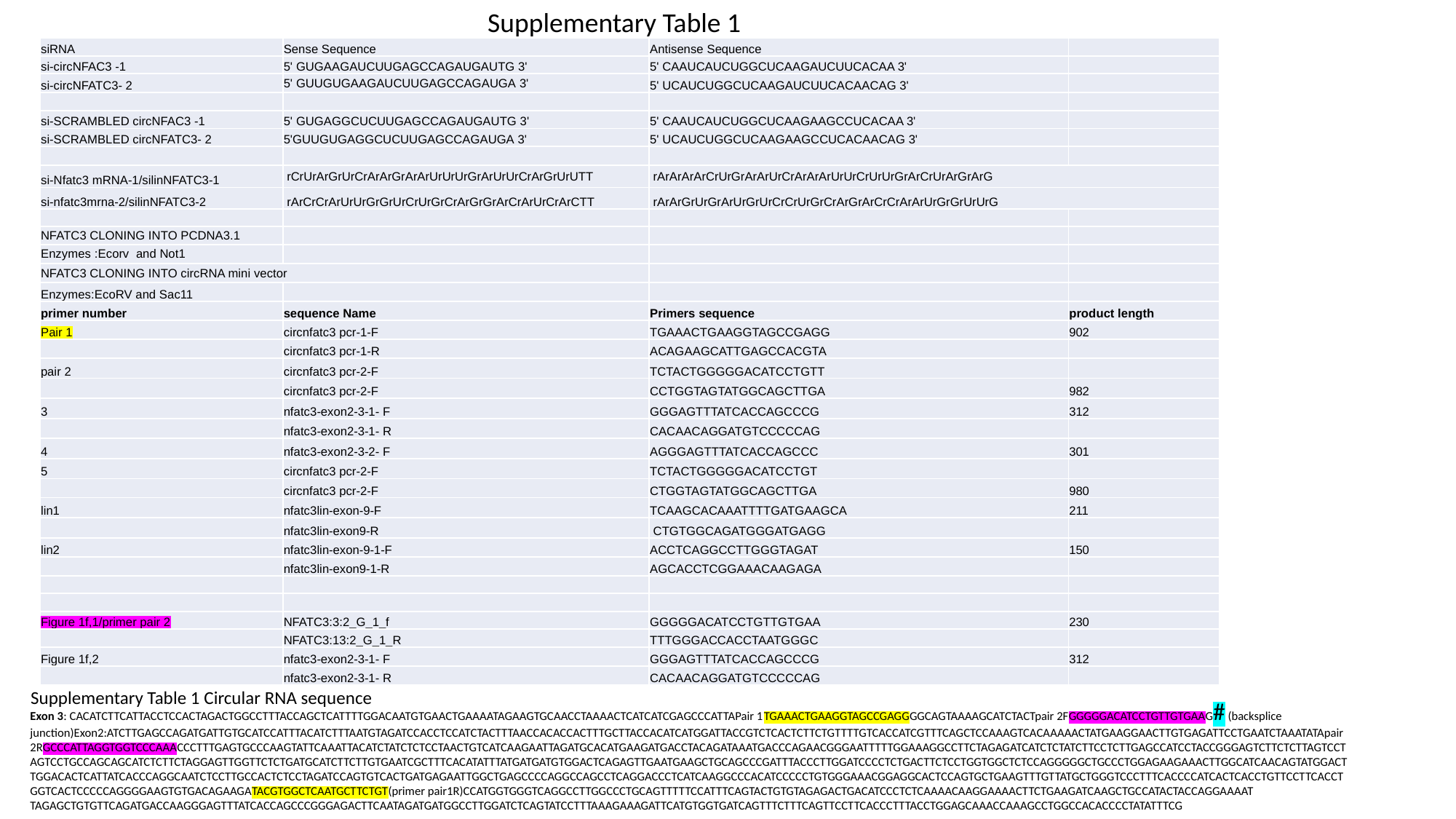

Supplementary Table 1
| siRNA | Sense Sequence | Antisense Sequence | |
| --- | --- | --- | --- |
| si-circNFAC3 -1 | 5' GUGAAGAUCUUGAGCCAGAUGAUTG 3' | 5' CAAUCAUCUGGCUCAAGAUCUUCACAA 3' | |
| si-circNFATC3- 2 | 5' GUUGUGAAGAUCUUGAGCCAGAUGA 3' | 5' UCAUCUGGCUCAAGAUCUUCACAACAG 3' | |
| | | | |
| si-SCRAMBLED circNFAC3 -1 | 5' GUGAGGCUCUUGAGCCAGAUGAUTG 3' | 5' CAAUCAUCUGGCUCAAGAAGCCUCACAA 3' | |
| si-SCRAMBLED circNFATC3- 2 | 5'GUUGUGAGGCUCUUGAGCCAGAUGA 3' | 5' UCAUCUGGCUCAAGAAGCCUCACAACAG 3' | |
| | | | |
| si-Nfatc3 mRNA-1/silinNFATC3-1 | rCrUrArGrUrCrArArGrArArUrUrUrGrArUrUrCrArGrUrUTT | rArArArArCrUrGrArArUrCrArArArUrUrCrUrUrGrArCrUrArGrArG | |
| si-nfatc3mrna-2/silinNFATC3-2 | rArCrCrArUrUrGrGrUrCrUrGrCrArGrGrArCrArUrCrArCTT | rArArGrUrGrArUrGrUrCrCrUrGrCrArGrArCrCrArArUrGrGrUrUrG | |
| | | | |
| NFATC3 CLONING INTO PCDNA3.1 | | | |
| Enzymes :Ecorv and Not1 | | | |
| NFATC3 CLONING INTO circRNA mini vector | | | |
| Enzymes:EcoRV and Sac11 | | | |
| primer number | sequence Name | Primers sequence | product length |
| Pair 1 | circnfatc3 pcr-1-F | TGAAACTGAAGGTAGCCGAGG | 902 |
| | circnfatc3 pcr-1-R | ACAGAAGCATTGAGCCACGTA | |
| pair 2 | circnfatc3 pcr-2-F | TCTACTGGGGGACATCCTGTT | |
| | circnfatc3 pcr-2-F | CCTGGTAGTATGGCAGCTTGA | 982 |
| 3 | nfatc3-exon2-3-1- F | GGGAGTTTATCACCAGCCCG | 312 |
| | nfatc3-exon2-3-1- R | CACAACAGGATGTCCCCCAG | |
| 4 | nfatc3-exon2-3-2- F | AGGGAGTTTATCACCAGCCC | 301 |
| 5 | circnfatc3 pcr-2-F | TCTACTGGGGGACATCCTGT | |
| | circnfatc3 pcr-2-F | CTGGTAGTATGGCAGCTTGA | 980 |
| lin1 | nfatc3lin-exon-9-F | TCAAGCACAAATTTTGATGAAGCA | 211 |
| | nfatc3lin-exon9-R | CTGTGGCAGATGGGATGAGG | |
| lin2 | nfatc3lin-exon-9-1-F | ACCTCAGGCCTTGGGTAGAT | 150 |
| | nfatc3lin-exon9-1-R | AGCACCTCGGAAACAAGAGA | |
| | | | |
| | | | |
| Figure 1f,1/primer pair 2 | NFATC3:3:2\_G\_1\_f | GGGGGACATCCTGTTGTGAA | 230 |
| | NFATC3:13:2\_G\_1\_R | TTTGGGACCACCTAATGGGC | |
| Figure 1f,2 | nfatc3-exon2-3-1- F | GGGAGTTTATCACCAGCCCG | 312 |
| | nfatc3-exon2-3-1- R | CACAACAGGATGTCCCCCAG | |
Supplementary Table 1 Circular RNA sequence
Exon 3: CACATCTTCATTACCTCCACTAGACTGGCCTTTACCAGCTCATTTTGGACAATGTGAACTGAAAATAGAAGTGCAACCTAAAACTCATCATCGAGCCCATTAPair 1TGAAACTGAAGGTAGCCGAGGGGCAGTAAAAGCATCTACTpair 2FGGGGGACATCCTGTTGTGAAG# (backsplice junction)Exon2:ATCTTGAGCCAGATGATTGTGCATCCATTTACATCTTTAATGTAGATCCACCTCCATCTACTTTAACCACACCACTTTGCTTACCACATCATGGATTACCGTCTCACTCTTCTGTTTTGTCACCATCGTTTCAGCTCCAAAGTCACAAAAACTATGAAGGAACTTGTGAGATTCCTGAATCTAAATATApair2RGCCCATTAGGTGGTCCCAAACCCTTTGAGTGCCCAAGTATTCAAATTACATCTATCTCTCCTAACTGTCATCAAGAATTAGATGCACATGAAGATGACCTACAGATAAATGACCCAGAACGGGAATTTTTGGAAAGGCCTTCTAGAGATCATCTCTATCTTCCTCTTGAGCCATCCTACCGGGAGTCTTCTCTTAGTCCTAGTCCTGCCAGCAGCATCTCTTCTAGGAGTTGGTTCTCTGATGCATCTTCTTGTGAATCGCTTTCACATATTTATGATGATGTGGACTCAGAGTTGAATGAAGCTGCAGCCCGATTTACCCTTGGATCCCCTCTGACTTCTCCTGGTGGCTCTCCAGGGGGCTGCCCTGGAGAAGAAACTTGGCATCAACAGTATGGACTTGGACACTCATTATCACCCAGGCAATCTCCTTGCCACTCTCCTAGATCCAGTGTCACTGATGAGAATTGGCTGAGCCCCAGGCCAGCCTCAGGACCCTCATCAAGGCCCACATCCCCCTGTGGGAAACGGAGGCACTCCAGTGCTGAAGTTTGTTATGCTGGGTCCCTTTCACCCCATCACTCACCTGTTCCTTCACCTGGTCACTCCCCCAGGGGAAGTGTGACAGAAGATACGTGGCTCAATGCTTCTGT(primer pair1R)CCATGGTGGGTCAGGCCTTGGCCCTGCAGTTTTTCCATTTCAGTACTGTGTAGAGACTGACATCCCTCTCAAAACAAGGAAAACTTCTGAAGATCAAGCTGCCATACTACCAGGAAAAT
TAGAGCTGTGTTCAGATGACCAAGGGAGTTTATCACCAGCCCGGGAGACTTCAATAGATGATGGCCTTGGATCTCAGTATCCTTTAAAGAAAGATTCATGTGGTGATCAGTTTCTTTCAGTTCCTTCACCCTTTACCTGGAGCAAACCAAAGCCTGGCCACACCCCTATATTTCG

## Slide 2
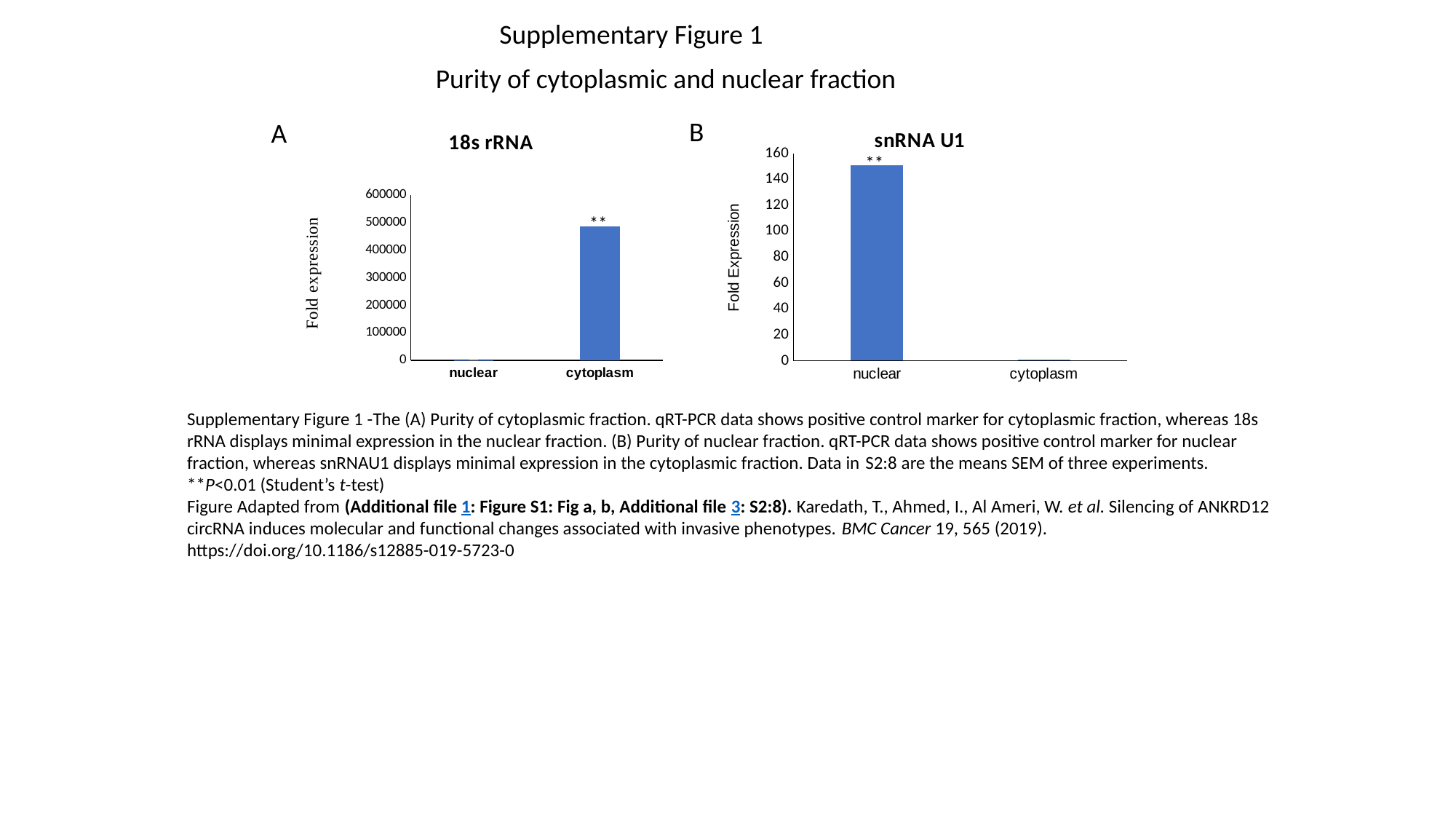

Supplementary Figure 1
Purity of cytoplasmic and nuclear fraction
B
A
### Chart:
| Category | snRNA U1 |
|---|---|
| nuclear | 150.8116236603091 |
| cytoplasm | 1.0 |
### Chart: 18s rRNA
| Category | 18srRNA |
|---|---|
| nuclear | 1.0 |
| cytoplasm | 487103.3334571714 |**
**
Fold Expression
Supplementary Figure 1 -The (A) Purity of cytoplasmic fraction. qRT-PCR data shows positive control marker for cytoplasmic fraction, whereas 18s rRNA displays minimal expression in the nuclear fraction. (B) Purity of nuclear fraction. qRT-PCR data shows positive control marker for nuclear fraction, whereas snRNAU1 displays minimal expression in the cytoplasmic fraction. Data in S2:8 are the means SEM of three experiments. **P<0.01 (Student’s t-test)
Figure Adapted from (Additional file 1: Figure S1: Fig a, b, Additional file 3: S2:8). Karedath, T., Ahmed, I., Al Ameri, W. et al. Silencing of ANKRD12 circRNA induces molecular and functional changes associated with invasive phenotypes. BMC Cancer 19, 565 (2019). https://doi.org/10.1186/s12885-019-5723-0

## Slide 3
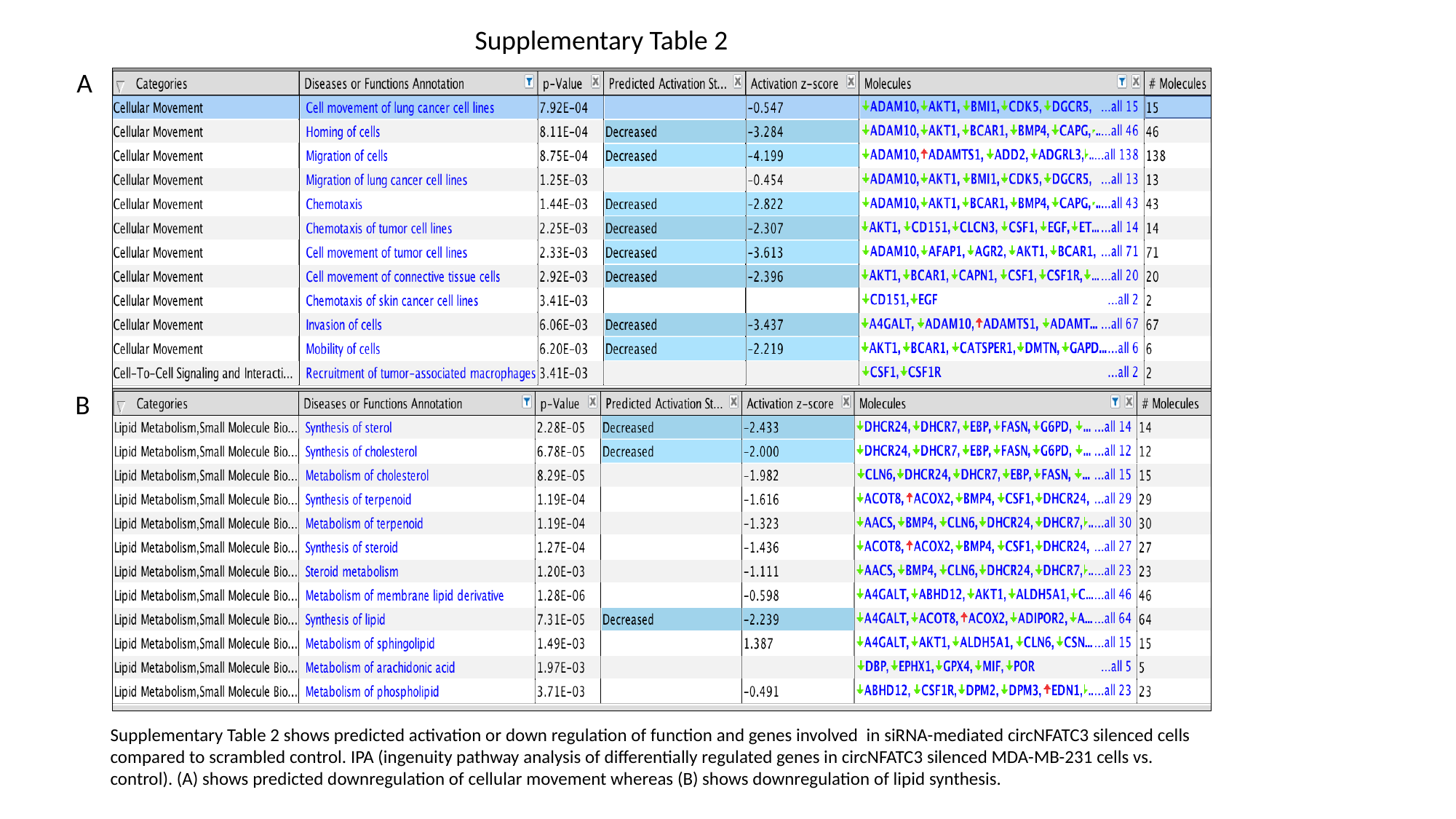

Supplementary Table 2
A
B
Supplementary Table 2 shows predicted activation or down regulation of function and genes involved in siRNA-mediated circNFATC3 silenced cells compared to scrambled control. IPA (ingenuity pathway analysis of differentially regulated genes in circNFATC3 silenced MDA-MB-231 cells vs. control). (A) shows predicted downregulation of cellular movement whereas (B) shows downregulation of lipid synthesis.

## Slide 4
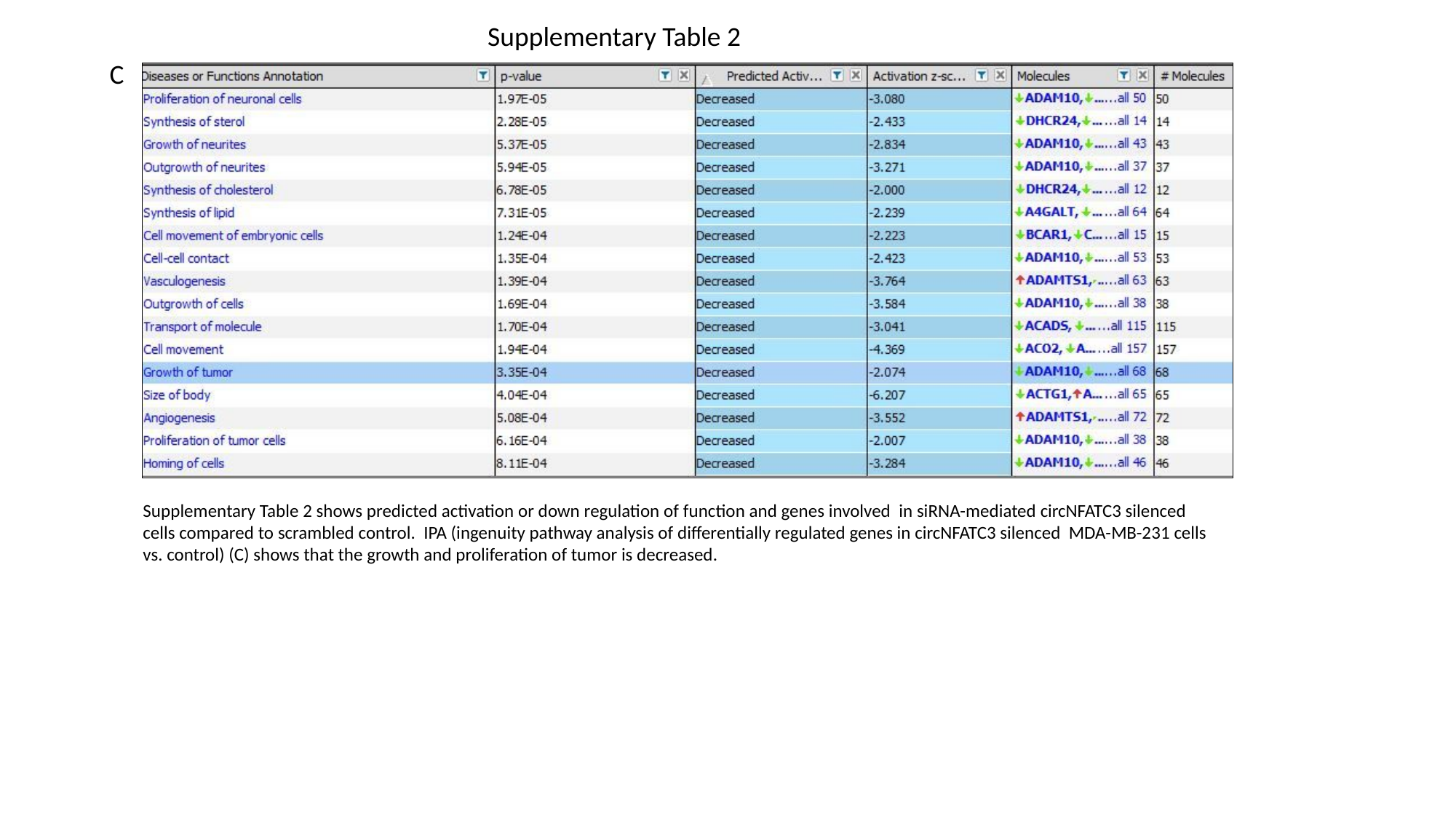

Supplementary Table 2
C
Supplementary Table 2 shows predicted activation or down regulation of function and genes involved in siRNA-mediated circNFATC3 silenced cells compared to scrambled control. IPA (ingenuity pathway analysis of differentially regulated genes in circNFATC3 silenced MDA-MB-231 cells vs. control) (C) shows that the growth and proliferation of tumor is decreased.

## Slide 5
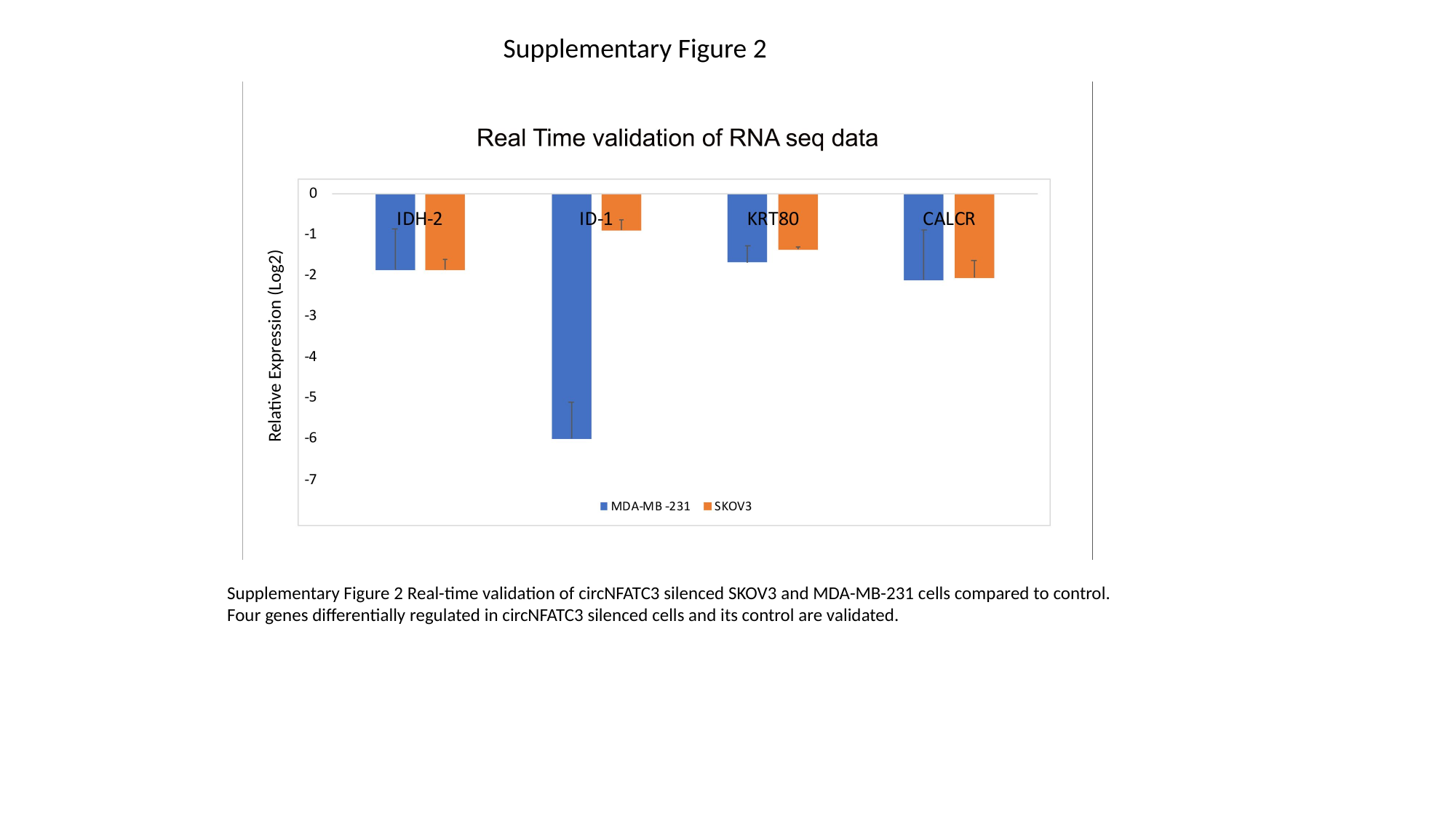

Supplementary Figure 2
Relative Expression (Log2)
Supplementary Figure 2 Real-time validation of circNFATC3 silenced SKOV3 and MDA-MB-231 cells compared to control. Four genes differentially regulated in circNFATC3 silenced cells and its control are validated.

## Slide 6
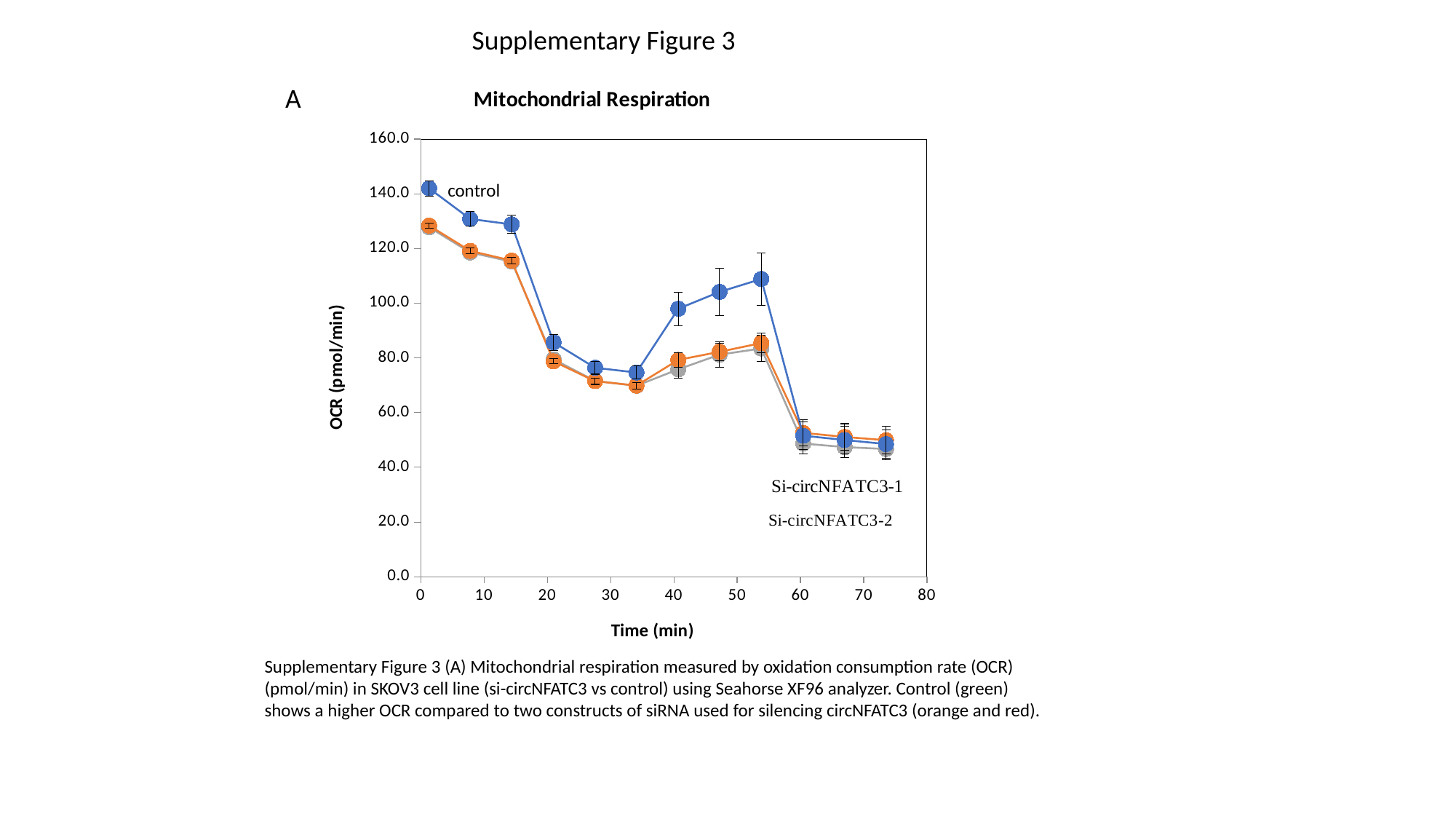

Supplementary Figure 3
### Chart: Mitochondrial Respiration
| Category | Group 1 | Group 2 | Group 3 | Unselected |
|---|---|---|---|---|A
control
Supplementary Figure 3 (A) Mitochondrial respiration measured by oxidation consumption rate (OCR) (pmol/min) in SKOV3 cell line (si-circNFATC3 vs control) using Seahorse XF96 analyzer. Control (green) shows a higher OCR compared to two constructs of siRNA used for silencing circNFATC3 (orange and red).

## Slide 7
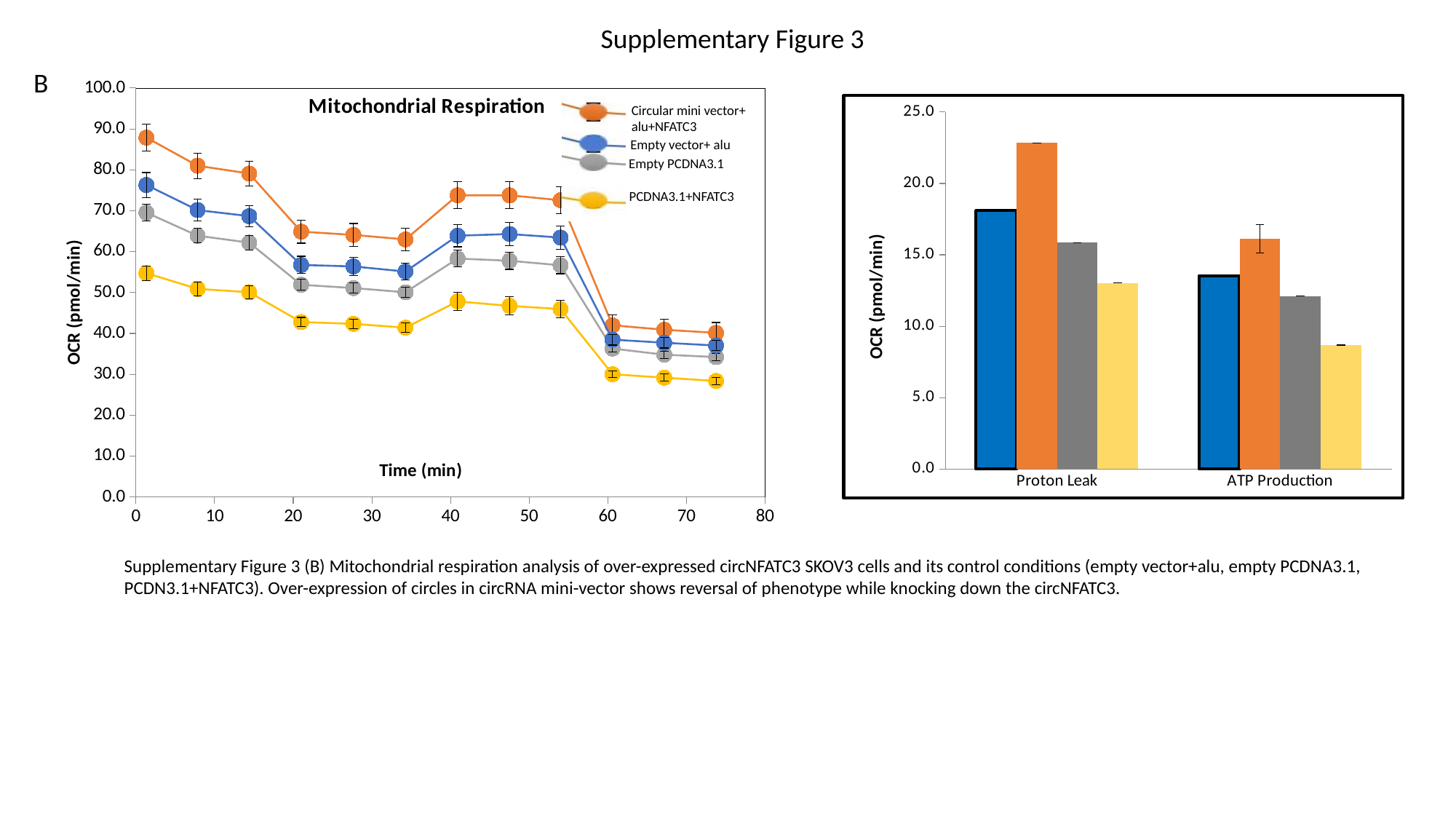

Supplementary Figure 3
B
### Chart: Mitochondrial Respiration
| Category | Group 1 | Group 2 | Group 3 | Group 4 | Group 5 | Unselected |
|---|---|---|---|---|---|---|
### Chart
| Category | Group 1 | Group 2 | Group 3 | Group 4 |
|---|---|---|---|---|
| Proton Leak | 18.101961135864258 | 22.848114013671875 | 15.853611946105957 | 13.026777267456055 |
| ATP Production | 13.542154312133789 | 16.108840942382812 | 12.119064331054688 | 8.678299903869629 |Circular mini vector+ alu+NFATC3
Empty vector+ alu
Empty PCDNA3.1
PCDNA3.1+NFATC3
Supplementary Figure 3 (B) Mitochondrial respiration analysis of over-expressed circNFATC3 SKOV3 cells and its control conditions (empty vector+alu, empty PCDNA3.1, PCDN3.1+NFATC3). Over-expression of circles in circRNA mini-vector shows reversal of phenotype while knocking down the circNFATC3.

## Slide 8
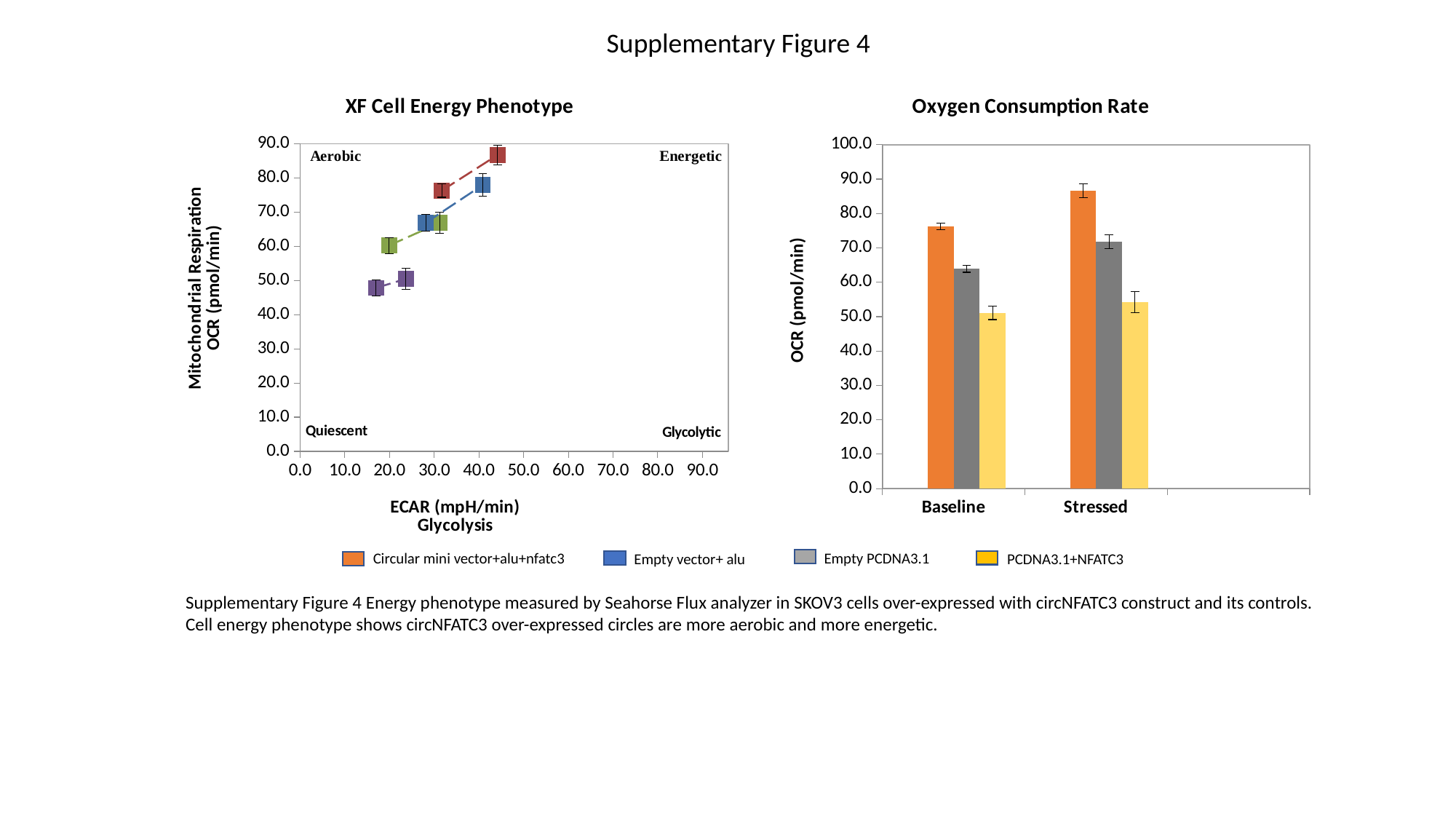

Supplementary Figure 4
### Chart: Oxygen Consumption Rate
| Category | Group 1 | Group 2 | Group 3 | Group 4 |
|---|---|---|---|---|
| Baseline | 64.5655288696289 | 76.29403686523438 | 63.912593841552734 | 51.145511627197266 |
| Stressed | 75.26549530029297 | 86.67937469482422 | 71.7542953491211 | 54.249671936035156 |
### Chart: XF Cell Energy Phenotype
| Category | | | | | |
|---|---|---|---|---|---|Empty PCDNA3.1
Circular mini vector+alu+nfatc3
Empty vector+ alu
PCDNA3.1+NFATC3
Supplementary Figure 4 Energy phenotype measured by Seahorse Flux analyzer in SKOV3 cells over-expressed with circNFATC3 construct and its controls. Cell energy phenotype shows circNFATC3 over-expressed circles are more aerobic and more energetic.

## Slide 9
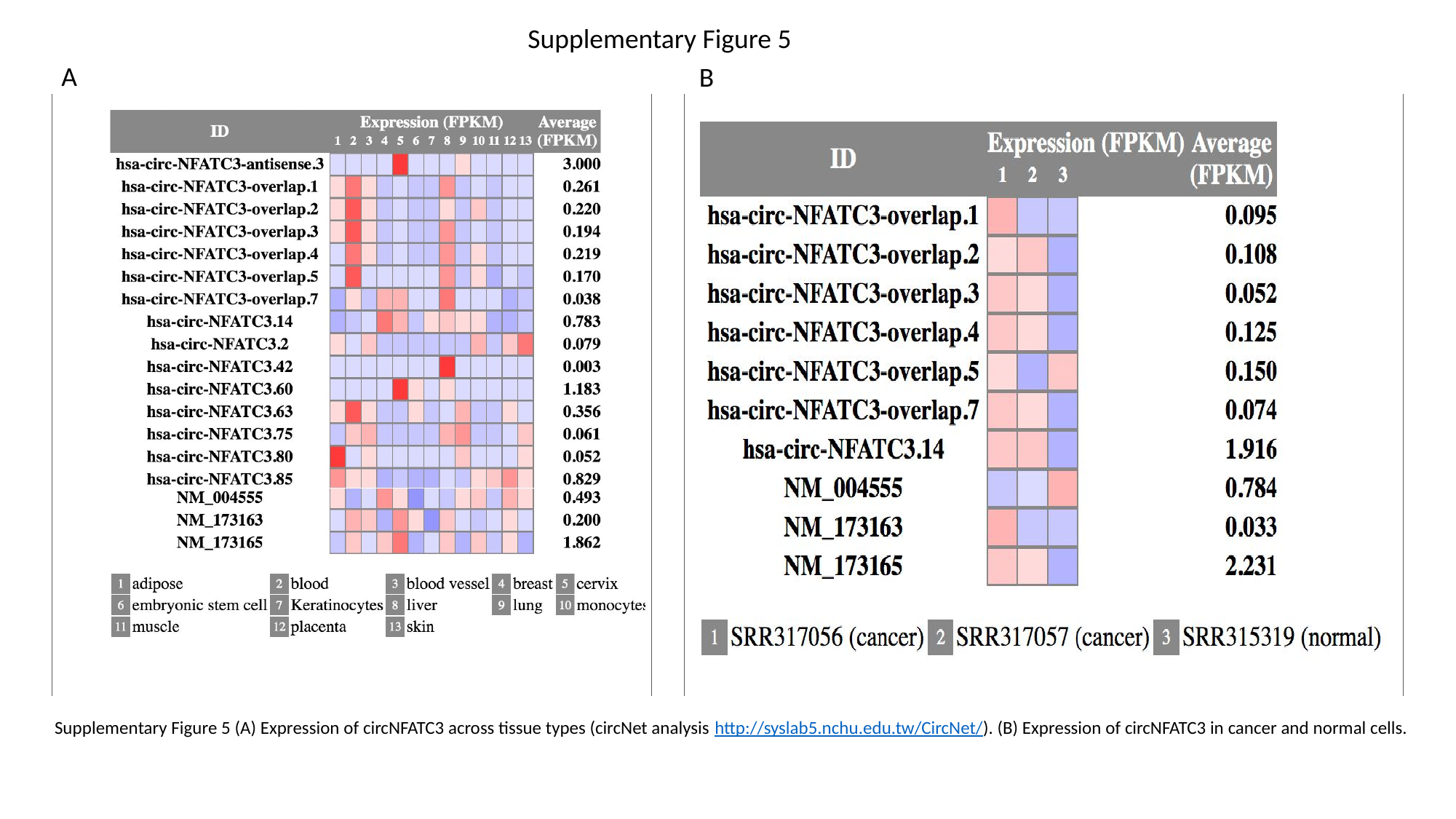

Supplementary Figure 5
A
B
Supplementary Figure 5 (A) Expression of circNFATC3 across tissue types (circNet analysis http://syslab5.nchu.edu.tw/CircNet/). (B) Expression of circNFATC3 in cancer and normal cells.

## Slide 10
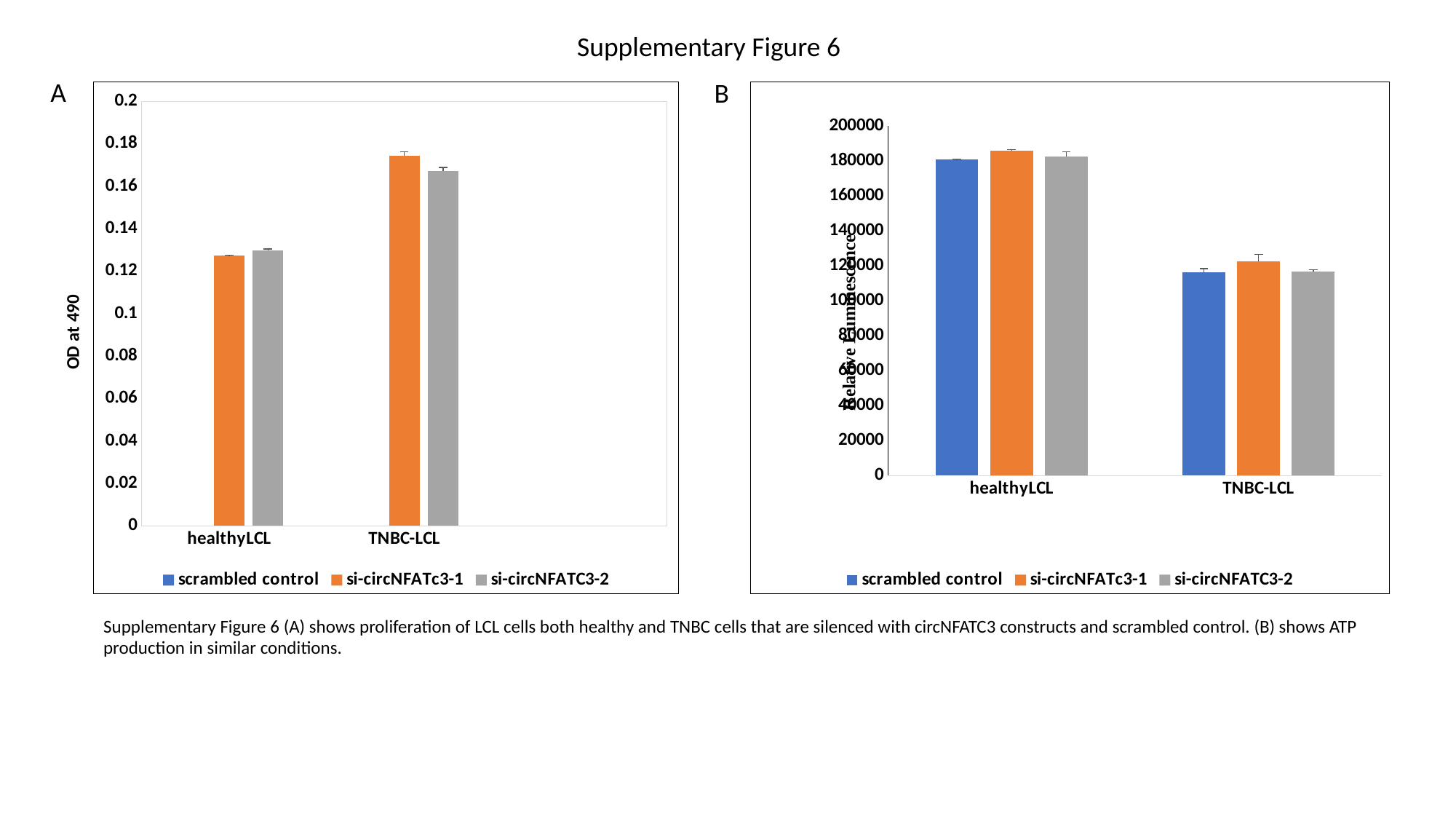

Supplementary Figure 6
A
B
### Chart
| Category | scrambled control | si-circNFATc3-1 | si-circNFATC3-2 |
|---|---|---|---|
| healthyLCL | 0.12574999999999997 | 0.12728125 | 0.12965625 |
| TNBC-LCL | 0.17678125 | 0.1745 | 0.16724999999999998 |
### Chart
| Category | scrambled control | si-circNFATc3-1 | si-circNFATC3-2 |
|---|---|---|---|
| healthyLCL | 181013.25 | 185888.5 | 182344.9696969697 |
| TNBC-LCL | 116487.20833333334 | 122745.76190476191 | 116765.28571428571 |OD at 490
Supplementary Figure 6 (A) shows proliferation of LCL cells both healthy and TNBC cells that are silenced with circNFATC3 constructs and scrambled control. (B) shows ATP production in similar conditions.

## Slide 11
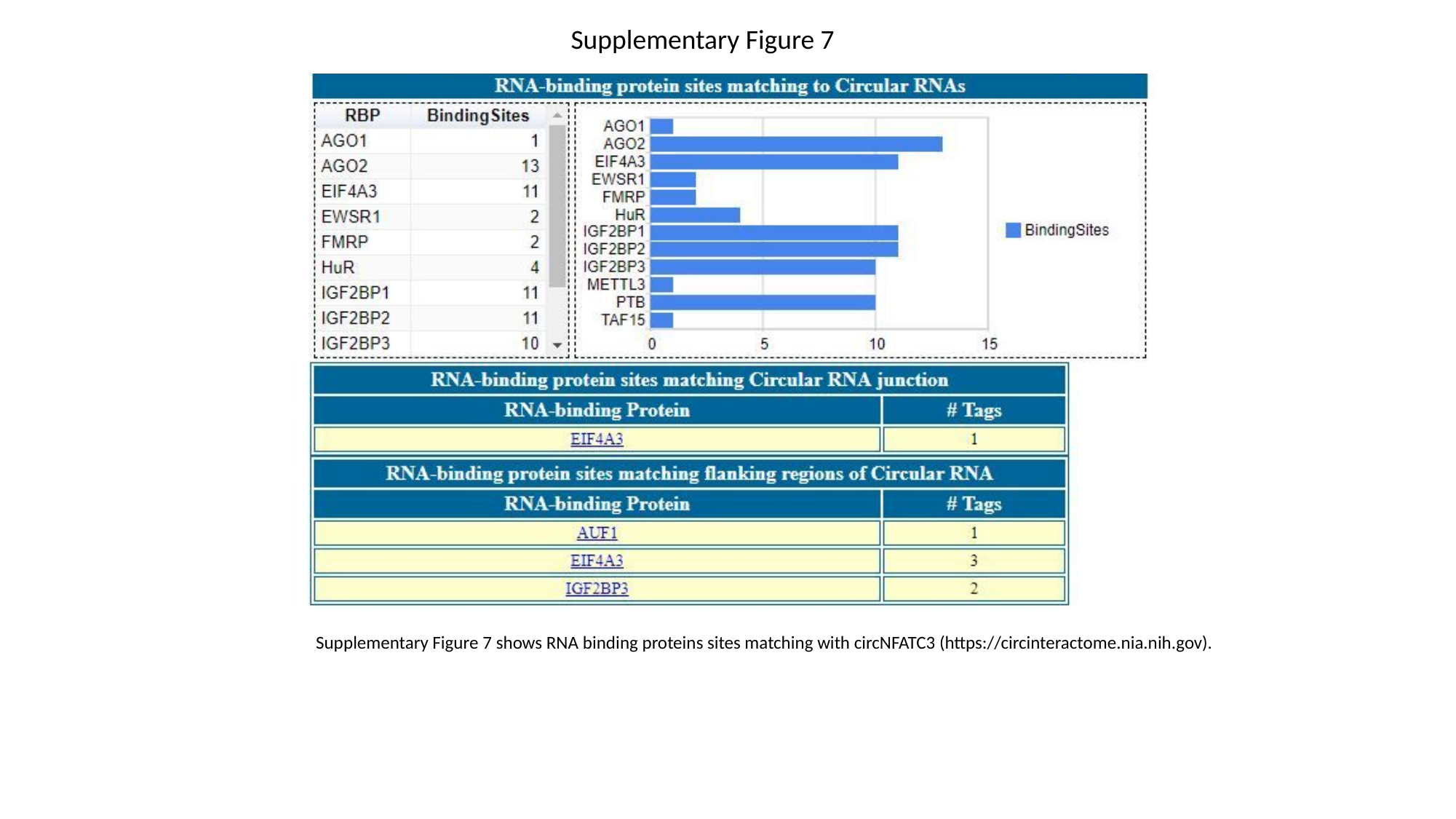

Supplementary Figure 7
Supplementary Figure 7 shows RNA binding proteins sites matching with circNFATC3 (https://circinteractome.nia.nih.gov).

## Slide 12
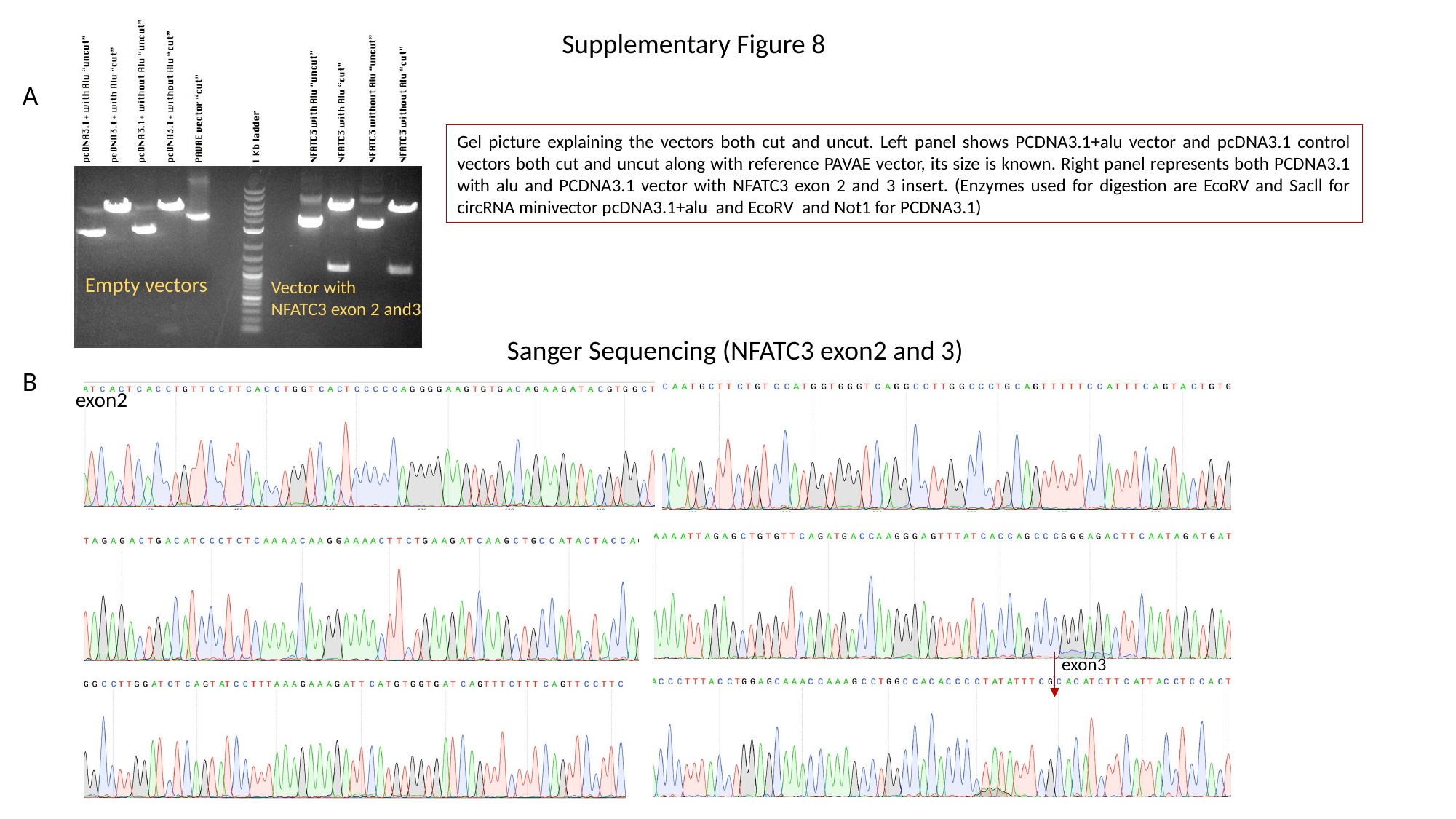

Supplementary Figure 8
A
Gel picture explaining the vectors both cut and uncut. Left panel shows PCDNA3.1+alu vector and pcDNA3.1 control vectors both cut and uncut along with reference PAVAE vector, its size is known. Right panel represents both PCDNA3.1 with alu and PCDNA3.1 vector with NFATC3 exon 2 and 3 insert. (Enzymes used for digestion are EcoRV and Sacll for circRNA minivector pcDNA3.1+alu and EcoRV and Not1 for PCDNA3.1)
Empty vectors
Vector with
NFATC3 exon 2 and3
Sanger Sequencing (NFATC3 exon2 and 3)
B
exon2
exon3

## Slide 13
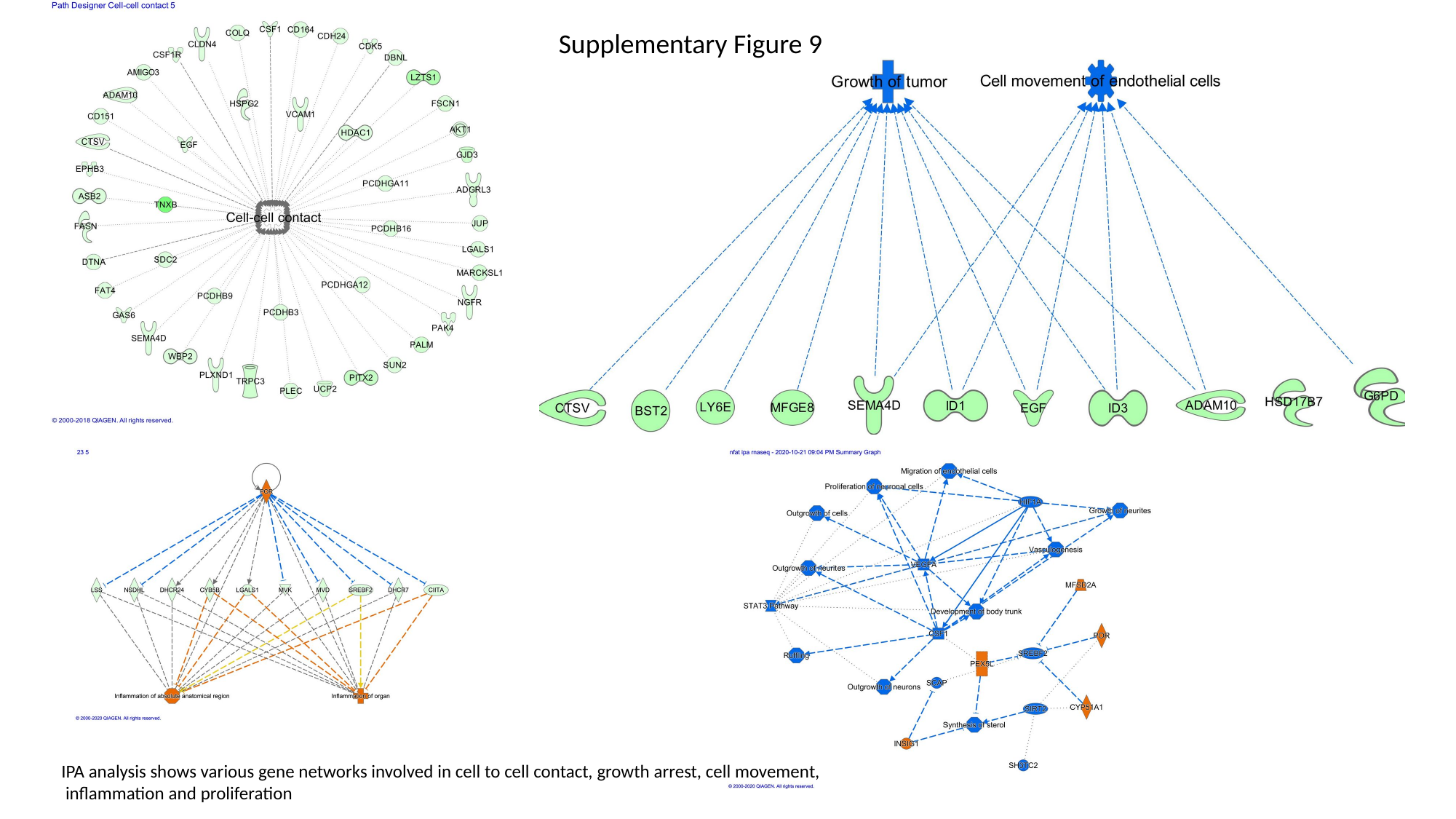

Supplementary Figure 9
IPA analysis shows various gene networks involved in cell to cell contact, growth arrest, cell movement,
 inflammation and proliferation
